# Beyond buzz‐pollination – departures from an adaptive plateau lead to new pollination syndromes

**DOI:** 10.1111/nph.15468

**Published:** 2018-10-12

**Authors:** Agnes S. Dellinger, Marion Chartier, Diana Fernández‐Fernández, Darin S. Penneys, Marcela Alvear, Frank Almeda, Fabián A. Michelangeli, Yannick Staedler, W. Scott Armbruster, Jürg Schönenberger

**Affiliations:** ^1^ Department of Botany and Biodiversity Research University of Vienna Rennweg 14 1030 Vienna Austria; ^2^ Herbario Nacional del Ecuador (QCNE) Instituto Nacional de Biodiversidad Río Coca E06‐115 e Isla Fernandina Quito Ecuador; ^3^ Department of Biology and Marine Biology University of North Carolina Wilmington 601 S. College Road Wilmington NC 28403 USA; ^4^ Institute of Biodiversity Science and Sustainability California Academy of Sciences 55 Music Concourse Drive San Francisco CA 94118‐4503 USA; ^5^ Institute of Systematic Botany The New York Botanical Garden 2900 Southern Blvd Bronx NY 10458‐5126 USA; ^6^ School of Biological Science University of Portsmouth King Henry 1 Street Portsmouth P01 2DY UK; ^7^ Institute of Arctic Biology University of Alaska Fairbanks Fairbanks AK 99775 USA

**Keywords:** buzz‐pollination, floral evolution, morphospace, pollinator shifts, vertebrate pollination

## Abstract

- Pollination syndromes describe recurring adaptation to selection imposed by distinct pollinators. We tested for pollination syndromes in Merianieae (Melastomataceae), which contain bee‐ (buzz‐), hummingbird‐, flowerpiercer‐, passerine‐, bat‐ and rodent‐pollinated species. Further, we explored trait changes correlated with the repeated shifts away from buzz‐pollination, which represents an ‘adaptive plateau’ in Melastomataceae.
- We used random forest analyses to identify key traits associated with the different pollinators of 19 Merianieae species and estimated the pollination syndromes of 42 more species. We employed morphospace analyses to compare the morphological diversity (disparity) among syndromes.
- We identified three pollination syndromes (‘buzz‐bee’, ‘mixed‐vertebrate’ and ‘passerine’), characterized by different pollen expulsion mechanisms and reward types, but not by traditional syndrome characters. Further, we found that ‘efficiency’ rather than ‘attraction’ traits were important for syndrome circumscription. Contrary to syndrome theory, our study supports the pooling of different pollinators (hummingbirds, bats, rodents and flowerpiercers) into the ‘mixed‐vertebrate’ syndrome, and we found that disparity was highest in the ‘buzz‐bee’ syndrome.
- We conclude that the highly adaptive buzz‐pollination system may have prevented shifts towards classical pollination syndromes, but provided the starting point for the evolution of a novel set of distinct syndromes, all having retained multifunctional stamens that provide pollen expulsion, reward and attraction.

## Abstract 2

See also the Commentary on this article by https://doi.org/10.1111/nph.15474.

## Introduction

The observation of recurring floral phenotypes associated with distinct pollinator groups has given rise to the concept of pollination syndromes (Delpino, 1890; Vogel, 1954; Stebbins, 1970; Faegri & van der Pijl, 1979; Endress, 1996). Pollinators are grouped into functional categories, i.e. groups of animals probably exerting similar selective pressures on flowers as a result of shared morphology, foraging behaviour/preferences and sensory abilities (Fenster *et al*., 2004). Thus, flowers pollinated by the same functional group of pollinators are expected to converge onto similar phenotypes in response to selection imposed by the most effective pollinators (defined as the product of visitation frequency and pollen transfer efficiency; e.g. Armbruster, 1988; Ne'eman *et al*., 2010; Ashworth *et al*., 2015; Fenster *et al*., 2015). Although a large body of literature reports pollination syndromes for certain plant lineages (Lázaro *et al*., 2008; Armbruster *et al*., 2011; Lagomarsino *et al*., 2017), and a recent quantitative evaluation of the concept found strong support even across angiosperms (Rosas‐Guerrero *et al*., 2014), other studies have raised concerns about the utility of the concept (e.g. Waser *et al*., 1996; Kingston & McQuillan, 2000; Ollerton *et al*., 2009). Major points of criticism include an over‐simplification of complex plant–animal interactions, a lack of clear terminology and difficulties in making comparisons across different taxonomic levels (summarized by Ollerton *et al*., 2009). Not all ‘classical’ traits (e.g. red coloration in bird syndrome, musty odour in bat syndrome) are necessarily equally selected for in all systems or geographical regions (Rosas‐Guerrero *et al*., 2014). Besides selection generated by pollinator effectiveness, the evolution of floral traits may also be mediated by antagonistic interactions (e.g. red coloration as bee avoidance in hummingbird flowers; Papiorek *et al*., 2014), competition for pollinators (e.g. colour variation in hummingbird‐pollinated Iochrominae; Muchhala *et al*., 2014) or the evolutionary history of the clade, and the developmental constraints embedded therein (e.g. constraints of possible floral trait combinations; Smith & Rausher, 2008; O'Meara *et al*., 2016). These interactions may lead to narrower, clade‐specific syndromes (e.g. Pérez *et al*., 2006; Johnson, 2013; Serrano‐Serrano *et al*., 2017).

Classical pollination syndromes are conceptually interpreted as systems specialized on only one (‘most effective’) functional group of pollinators, although it has long been recognized that additional secondary (less effective) pollinators are common (e.g. Rosas‐Guerrero *et al*., 2014; Ashworth *et al*., 2015). Indeed, Rosas‐Guerrero *et al*. (2014) showed that there is a non‐random association of pollination syndromes (e.g. bee–hummingbird, hummingbird–bat) and that ancestral pollinators are often retained as secondary pollinators as long as they do not incur a fitness cost (see also Aigner, 2006).

Finally, syndromes should capture adaptations for how to ‘attract and utilize’ (Fenster *et al*., 2004) pollinators. Many existing studies focus on a reduced set of traits primarily from the ‘attraction’ component (e.g. colour, reward and scent). This is particularly troublesome as the literature suggests stronger selection on the ‘utilization’ component (fitted with the pollinator to ensure pollen transfer, ‘efficiency function traits’; Ordano *et al*., 2008; Rosas‐Guerrero *et al*., 2011). Thus, it is timely to take a novel approach to pollination syndrome studies. Here, we integrate pollinator observations and floral trait data on both ‘classical’ syndrome traits and any trait that may be relevant for our study system (‘bottom up’ approach outlined by Ollerton *et al*., 2009), and use multivariate analyses to detect convergent associations between flower traits and pollinators (‘top down’ approach; Ollerton *et al*., 2009).

Buzz‐pollination by bees has evolved independently in many angiosperm lineages (found in at least 72 families) and is present in *c*. 22 000 species (Cardinal *et al*., 2018). A typical buzz‐pollinated flower is characterized by poricidal anthers, lack of nectar and pollen being the sole reward offered to pollinating bees (Buchmann, 1983). The functional group of ‘buzzing bees’ is taxonomically and morphologically highly diverse, as bees from at least 74 genera (seven families) are capable of producing distinct high‐frequency vibrations (‘buzz’) (de Luca & Vallejo‐Marín, 2013; Cardinal *et al*., 2018). The buzz‐pollination syndrome is not evenly distributed across angiosperms, however; whilst some lineages contain only a few species adapted for buzz‐pollination, some genera, such as *Solanum*, and families, such as Melastomataceae, show a conspicuous predominance of buzz‐pollination. In the latter, an estimated 98% of the *c*. 5000 species are buzz‐pollinated (Renner, 1989; Berger *et al*., 2016). Evolutionary success has been proposed as an explanation for the prevalence of buzz‐pollination in Melastomataceae, balancing the majority of species on an ‘adaptive peak’ (Macior, 1971). Given the considerable floral disparity (morphological diversity) amongst buzz‐bee‐pollinated Melastomataceae (e.g. genus *Leandra*; Reginato & Michelangeli, 2016), it is probably more appropriate to speak of an ‘adaptive plateau’ on which the family is wandering. Interestingly, recent studies have reported various departures from the buzz‐pollination syndrome to alternative pollinators (flies, wasps, hummingbirds, bats, passerines and rodents) in Melastomataceae (Lumer, 1980; Renner, 1989; Vogel, 1997; Dellinger *et al*., 2014; Brito *et al*., 2017). Although not yet formally tested, these shifts seem to be associated with complex changes in reward type (from pollen to nectar; Varassin *et al*., 2008 or to food bodies; Dellinger *et al*., 2014) or pollen expulsion mechanisms (e.g. from buzzing to a bellows mechanism; Dellinger *et al*., 2014). As buzz‐pollinated flowers represent a functionally highly complex, specialized pollination system very distinct from the majority of bee pollination systems, an understanding of trait combinations and associated new syndromes derived therefrom is particularly interesting.

Here, we analyse the floral morphology and pollination ecology of members of the Neotropical Melastomataceae tribe Merianieae (*c*. 300 species), which offers an ideal model system to investigate floral adaptations to different functional pollinator groups. Buzz‐pollination is clearly ancestral in Merianieae and independent shifts to different vertebrate pollination systems (including mixed hummingbird/bat and passerine pollination) have occurred repeatedly (Dellinger *et al*., 2014; see the Results section). We use state‐of‐the‐art statistical tools (random forests, Johnson, 2013; morphospaces, Chartier *et al*., 2017) to (1) describe the pollination syndromes (based on 61 floral traits) of 19 Merianieae species with known pollinators, (2) determine the respective roles of ‘classical’ pollination syndrome traits and Merianieae‐specific traits, and (3) predict pollinators for 42 species, for which pollinators have never been observed. This enables us to provide a broad understanding of the floral morphologies that characterize the ‘buzz’‐morphology as the evolutionary starting point in Merianieae, and to understand the floral trait changes that have occurred along the evolutionary paths away from the ‘buzz‐pollination plateau’ to different vertebrate pollination systems. Furthermore, by mapping pollination syndromes onto a phylogeny, we provide evidence that floral adaptations in Merianieae indeed represent convergences to different functional pollinator groups, as postulated under the pollination syndrome concept.

## Materials and Methods

### Taxon sampling and floral traits

We aimed to capture both the morphological and taxonomic diversity in Merianieae by selecting 61 species (*c*. 20% of Merianieae) from five of the eight currently recognized genera for our study. Flower material was collected throughout the distribution range of Merianieae (north to south from Costa Rica to Brazil, east to west from Antilles to Ecuador) and stored in 70% ethanol; details on sampling localities can be found in Supporting Information Table S1.

Based on earlier studies of pollination syndromes (e.g. Ollerton *et al*., 2009) and on floral morphology in Melastomataceae (e.g. Varassin *et al*., 2008; Mendoza‐Cifuentes & Fernández‐Alonso, 2010; Cotton *et al*., 2014; Dellinger *et al*., 2014), we have compiled a list of 61 floral characters potentially important for pollination (for the justification of character choice, see Notes S1). Our floral dataset is based on direct field observations, photographs, descriptions on herbarium sheet labels, scanning electron microscopy (SEM), light microscopy and high‐resolution X‐ray computed tomography (HR‐XCT). For SEM, flowers were dissected and, for each species, the hypanthium, one petal, two stamens and one style were prepared (for details on preparation, see Dellinger *et al*., 2014). For HR‐XCT, entire flowers or stamens of 57 species were placed into a contrasting agent (1% phosphotungstic acid–70% ethanol) for 4 wk and mounted for scanning by placing them into plastic cups (Semadeni Plastics Group, Ostermundigen, Switzerland) with acrylic pillow foam arranged around the samples to prevent them from moving during the scanning procedure (for details on the HR‐XCT methodology, see Staedler *et al*., 2013, 2018). Three‐dimensional models of flowers and stamens were reconstructed (XML‐Reconstructor) and visualized in the software amira; raw scan data have been deposited on the open source platform Phaidra (https://phaidra.univie.ac.at/).

### Pollinator observations

Pollinator information from the literature was available for eight species. In addition, we monitored pollinators using video cameras (Sony Camcorder, Tokyo, Japan) and direct observations at field sites in Ecuador (2016/2017) and Costa Rica (2015/2018) for 11 more species (Tables 1, S2). We filmed single inflorescences during daytime (06:00–18:00 h) and night monitored (18:00–00:00 h) five species. For each video, we replayed a minimum of three random 30‐min intervals using the software playmemorieshome (total average of 11.3 h of daytime and 8.2 h of night‐time observation per species). We scored visitors as pollinators if they caused pollen release from stamens and came into contact with stigmas. Floral visitors were classified as ‘buzz‐bee’, ‘hummingbird’, ‘bat’, ‘flowerpiercer’ (nectar‐consuming passerine birds), ‘passerine’ (in this study, including Thraupidae visiting flowers for non‐nectar rewards) and ‘rodent’ (Table 1). Bat and rodent visits to *Meriania* were recorded only during the night. This resulted in a total of 19 species with known pollinators in Merianieae. Of these species, six (*M. *aff*. sanguinea*,* M. furvanthera*,* M. phlomoides*,* M. pichinchensis*,* M. sanguinea* and *M. tomentosa*) are pollinated by two types of pollinators (e.g. diurnal hummingbirds and nocturnal bats, see Table 1) and would usually be classified into two different functional groups (e.g. Faegri & van der Pijl, 1979). In *Meriania*, these pollinators actually all visit flowers looking for the same reward (nectar). For the two other nectar‐producing species, *M. costata* and *M. quintuplinervis*, no nocturnal observations were made, but additional nocturnal pollinators (bats and/or rodents) cannot be ruled out. This lack of information must be treated with care in pollinator classification analyses (see next paragraph).

**Table 1:** Merianieae species with known pollinators, source of pollinator observation and syndrome estimation using random forest (RF) analyses for the ‘six‐syndrome model’ (‘buzz‐bee’, ‘hummingbird/?’, ‘hummingbird/bat’, ‘hummingbird/rodent’, ‘flowerpiercer/rodent’, ‘passerine’) and ‘three‐syndrome model’ (‘buzz‐bee’, ‘mixed‐vertebrate’, ‘passerine’)

Species	Confirmed pollinator group	Source of pollinator observation	Estimation ‘six‐syndrome model’	Estimation ‘three‐syndrome model’
*Adelobotrys adscendens* (Sw.) Triana	**Buzz‐bee**	A. S. Dellinger (pers. obs.)	**Buzz**‐**bee (0.94)**/passerine (0.06)	**Buzz**‐**bee (0.9)**/passerine (0.1)
*Graffenrieda cucullata* (Triana) L.O. Williams	**Buzz‐bee**	A. S. Dellinger (pers. obs.)	**Buzz**‐**bee (0.59)**/passerine (0.41)	Passerine (0.55)/**buzz**‐**bee (0.45)**
*Meriania drakei* (Cogn.) Wurdack	**Buzz‐bee**	A. S. Dellinger (pers. obs.)	**Buzz**‐**bee (1)**	**Buzz**‐**bee (1)**
*Meriania hernandoi* L. Uribe	**Buzz‐bee**	A. S. Dellinger (pers. obs.)	**Buzz**‐**bee (1)**	**Buzz**‐**bee (1)**
*Meriania longifolia* (Naudin) Cogn.	**Buzz‐bee**	Renner (1989)	**Buzz**‐**bee (1)**	**Buzz**‐**bee (1)**
*Meriania maguirei* Wurdack	**Buzz‐bee**	A. S. Dellinger (pers. obs.)	**Buzz**‐**bee (1)**	**Buzz**‐**bee (1)**
*Meriania maxima* Markgr.	**Buzz‐bee**	A. S. Dellinger (pers. obs.)	**Buzz**‐**bee (1)**	**Buzz**‐**bee (1)**
*Meriania furvanthera* Wurdack	**Flowerpiercer/rodent**	A. S. Dellinger (pers. obs.)	HB (0.67)/**FR (0)**	**MV (1)**
*Meriania costata* Wurdack	**Hummingbird/?**	A. S. Dellinger (pers. obs.)	HB (0.89)/**H/? (0)**	**MV (1)**
*Meriania quintuplinervis* Naudin	**Hummingbird/?**	E. Calderón‐Sáenz (unpublished)	HB (1)/**H/? (0)**	**MV (1)**
*Meriania pichinchensis* Wurdack	**Hummingbird/bat**	Muchhala & Jarrin‐V (2002); A. S. Dellinger (pers. obs.)	**HB (0.8)**/H (0.2)	**MV (1)**
*Meriania* aff. *sanguinea*	**Hummingbird/bat**	A. S. Dellinger (pers. obs.)	HR (1)/**HB (0)**	**MV (1)**
*Meriania phlomoides* (Triana) Almeda	**Hummingbird/bat**	Vogel (1997); A. S. Dellinger (pers. obs.)	**HB (0.84)**/H (0.16)	**MV (1)**
*Meriania tomentosa* (Cogn.) Wurdack	**Hummingbird/bat**	A. S. Dellinger (pers. obs.)	**HB (1)**	**MV (1)**
*Meriania sanguinea* Wurdack	**Hummingbird/rodent**	A. S. Dellinger (pers. obs.)	HB (1)/**HR (0)**	**MV (1)**
*Axinaea confusa* E. Cotton & Borchs.	**Passerine**	Dellinger *et al*. (2014)	**Passerine (1)**	**Passerine (1)**
*Axinaea costaricensis* Cogn.	**Passerine**	Dellinger *et al*. (2014)	**Passerine (1)**	**Passerine (1)**
*Axinaea macrophylla* (Naudin) Triana	**Passerine**	Rojas‐Nossa (2007)	**Passerine (1)**	**Passerine (1)**
*Axinaea sclerophylla* Triana	**Passerine**	Dellinger *et al*., 2014	**Passerine (1)**	**Passerine (1)**

The first and second most probable group assignments and estimation probabilities (0 (0%)–1 (100%)) are given for each species. The variable group assignment in buzz‐bee‐pollinated *A. adscendens* and *G. cucullata* is due to these flowers presenting highly distinct morphologies from all other buzz‐bee‐pollinated species with known pollinators, underpinning the diversity of the ‘buzz‐bee’ syndrome; misclassification is alleviated once more species with similar morphologies are included in syndrome estimation. ‘?’ indicates a lack of nocturnal pollinator observations. Abbreviations: H/?, ‘hummingbird/?’; HB, ‘hummingbird/bat’; HR, ‘hummingbird/rodent’; FR, ‘flowerpiercer/rodent’; MV, mixed‐vertebrate. Bold type indicates the correct pollination syndrome.

### Identification of floral characters differentiating pollinator groups

We used the statistical classification method of random forests (RF) to identify the most important floral characters differentiating functional pollinator groups in Merianieae with known pollinators (Breiman, 2001; for application in the same context, see Johnson, 2013). In RF analyses, a large number of decision trees are built on subsets of data by trying different variables at each node and assessing the quality of the specific variable in reducing the tree's entropy (i.e. power of character in splitting data into known classes). As only 63% of input data are used in each tree, the remaining out‐of‐bag (OOB) observations are used to estimate classification error and reduction in model accuracy when one character is removed (reduction in Gini index; Cutler *et al*., 2007). We ran two different models: (1) a ‘six‐syndrome model’ separating pollinators into six functional pollinator groups (‘buzz‐bee’, ‘hummingbird/?’, ‘hummingbird/bat’, ‘hummingbird/rodent’, ‘flowerpiercer/rodent’, ‘passerine’); and (2) a ‘three‐syndrome model’ separating pollinators into three functional groups (‘buzz‐bee’, ‘mixed‐vertebrate’ and ‘passerine’). The ‘mixed‐vertebrate’ group encompasses all nectar‐secreting Merianieae species where pollinators foraging for nectar cause pollen release when inserting tongues/bills/heads into flowers, and hence possibly selected for a common pollen expulsion mechanism (to compare the flower morphology of these species, see Fig. S1). We calculated 100 RFs of 500 trees each and seven variables tried at each split (mtry). The importance of each variable (floral character) in separating the pollinator groups was ranked by the mean decrease in Gini index over all 100 RFs. All analyses were run using the randomforest package 4.6‐12 (Liaw & Wiener, 2002) in R 3.3.0 (R Core Team, 2017).

### Estimation of pollinators

To estimate the pollinators of species for which no observations were available, we ran the function *predict* (stats) on the RFs previously trained with data from the 19 species with known pollinators (Table S2). As RFs cannot handle missing data, the variables ‘reward type’ (69.1% of data missing) and ‘pollen expulsion mechanism’ (95.2% of data missing) were removed from the dataset despite their importance (see Results). As the removal of characters with high predictive power may reduce model accuracy, we first ascertained that the error rates remained low by re‐running predictions of species with known pollinators on the reduced trait dataset (see Table S3). In 19 of the 42 species for which we predicted pollinators, additional characters included missing data. For these, we ran separate predictions excluding the characters with missing data (Table S4). Predictions from these separate runs were collated with the results obtained from the other runs. We ran predictions for the ‘three‐syndrome model’ only because the ‘six‐syndrome model’ failed to predict species with two pollinator types into separate syndromes (see Results). All predictions were run 100 times to account for possible inconsistencies in group assignment.

### Morphospace analyses and disparity

To understand the variation in morphological diversity (disparity), we constructed a morphospace from the full set of 61 floral characters. We grouped species into the three pollination syndromes (‘buzz‐bee’, ‘mixed‐vertebrate’ and ‘passerine) estimated from RF analyses (Table S4). A dissimilarity matrix (mean character difference *D* between each pair of taxa; Foote, 1999) was calculated following Chartier *et al*. (2017), whose approach allows the accommodation of all types of data (binary, categorical and continuous). Principal coordinates analyses (PCoAs) were calculated on the dissimilarity matrix to visualize morphospace occupation. A PERMANOVA was run on the dissimilarity matrix to test for morphological differences between pollination syndromes using the function *adonis* (vegan) (Oksanen *et al*., 2018) in R, with 10 000 permutations to calculate a pseudo *F*‐ratio. We estimated the disparity from the distance matrix as the mean pairwise dissimilarity ($\overset{¯}{D}$) for each pollination syndrome and compared among groups with a non‐parametric Kruskal–Wallis test. Partial disparity (partial contribution of each pollination system to total disparity) was calculated from the coordinates of each species in the morphospace following Foote (1993).

### Phylogeny and ancestral character estimation

To ascertain whether pollinator shifts in Merianieae have occurred repeatedly, and hence similar floral phenotypes indeed represent convergences to different pollinator groups as assumed under the concept of pollination syndromes, we used a trimmed phylogeny for the 61 Merianieae species included in this study. The presented phylogeny stems from larger phylogenetic analyses for the entire Merianieae, which will be discussed in detail elsewhere (F.A. Michelangeli *et al*., unpublished; for details, see Table S5). The expanded Merianiae phylogeny has 190 terminals representing 150 taxa of Merianieae and eight outgroups (four species of Miconieae, three species of *Physeterostemon* and one species of *Eriocnema*). Some species for which species boundaries are problematic are represented by more than one accession. Total genomic DNA was isolated from silica‐dried or herbarium material using the DNAeasy plant mini kit from Qiagen (Qiagen, Valencia, CA, USA) following the modifications suggested by Alexander *et al*. (2007) and Martin *et al*. (2008). Some samples were isolated using the cetyltrimethylammonium bromide (CTAB) method as modified by Doyle & Doyle (1987), scaled down for 600 μl of extraction buffer. The molecular dataset includes six loci markers, including two nuclear ribosomal loci (internal and external transcribed spacers, nrITS and nrETS) and four plastid loci (portions of the *ndhF* and *rbcL* genes and the intergenic spacers *accD‐psaI* and *psbK‐psbL*). All of these regions have been widely used in Melastomataceae systematics, and PCR primers and conditions follow Clausing & Renner (2001), Fritsch *et al*. (2004), Michelangeli *et al*. (2004, 2008, 2013), Martin *et al*. (2008), Reginato *et al*. (2010) and Kriebel *et al*. (2015). Cycle sequencing was performed with the same forward and reverse primers as used for amplification through the high‐throughput sequencing service of the University of Washington or Macrogen (Rockville, MD, USA). Sequence contigs were built with sequencher 4.9 (GeneCode Corp., Ann Arbor, MI, USA) or geneious v7.1.9. (Biomatters Ltd., Auckland, New Zealand). Sequence alignment was performed with muscle (Edgar, 2004) as implemented through the geneious plugin. Sequence evolution models for each locus were set to GTR. Separate phylogenetic analyses were conducted for each dataset using maximum likelihood (ML) in RaxML v. 8.2.10 (Stamatakis, 2014) and run through the cipres Science Gateway (http://www.phylo.org/; Miller *et al*., 2010). Rapid bootstrapping (BS) was performed on the ML tree using RaxML at 1000 replicates to determine branch support. Once we had ensured that there was no topological conflict among loci (BS threshold > 70), all loci were combined into a single matrix. ML was run on the combined matrix with six partitions maintaining the same parameters as above.

Ancestral states of pollination syndromes and three of the most important floral characters with data present for all species (Table 2: ‘appendage shape’ (as a proxy of ‘pollen expulsion mechanism’ and ‘reward type’), ‘filament ruptures’ (as a proxy of ‘reward type’), ‘relative position of stigma vs corolla opening’) were estimated using ML methods. For all four characters, models with ‘equal rates’ and ‘all rates different’ were run using the function *ace* (ape; Paradis *et al*., 2004) and a likelihood ratio test was subsequently performed to select the best‐fit model for each character. Stochastic character mapping (1000 iterations) with the empirical Bayes method on the optimal model was performed with the function *make.simmap* (phytools; Revell, 2009) to validate ML estimation.

**Table 2:** Twenty floral characters of Merianieae ranked by importance (mean decrease in model accuracy and Gini index) in separating the three pollination syndromes and mean decrease in accuracy per syndrome averaged for the 100 RFs; * indicates classical pollination syndrome characters; detailed information on the floral characters can be found in Supporting Information Notes S1 and S2

Floral characters ranked by importance	Mean decrease in model accuracy	Mean decrease in Gini index	‘Buzz‐bee’	‘Mixed‐vertebrate’	‘Passerine’
Mode of pollen expulsion	0.087	1.533	0.127	0.074	0.071
Reward type*	0.051	1.1	0.065	0.03	0.095
Relative position of stigma vs corolla opening*	0.056	0.942	0.043	0.084	0.041
Filament ruptures	0.055	0.881	0.041	0.078	0.045
Petal gloss*	0.047	0.753	0.037	0.068	0.033
Orientation of flower*	0.041	0.648	0.021	0.051	0.045
Corolla height*	0.022	0.604	0.042	0.022	0.005
Stigma shape	0.029	0.572	0.038	0.022	0.029
Pollen grain diameter	0.023	0.534	0.049	0	0.029
Relation corolla diameter : height	0.011	0.491	0.016	0.004	0.007
Corolla shape*	0.025	0.478	0.016	0.007	0.061
Structure of stamen appendage	0.018	0.468	0.002	0.028	0.029
Corolla shape change during anthesis	0.022	0.454	0.044	0.018	−0.002
Change of androecial arrangement during anthesis	0.021	0.397	0.008	0.034	0.026
Structure of adaxial thecal wall*	0.011	0.385	0.01	0.012	0.013
Level of anther pore*	0.017	0.372	0.01	0.022	0.019
Stamen appendage shape	0.009	0.283	0.002	0.008	0.027
Dimorphism in appendage volume	0.007	0.259	−0.003	0.005	0.025
Stigma diameter	0.007	0.225	−0.004	0.012	0.014
Style curvature	0.01	0.176	0.02	0	0.013

## Results

### Differentiation of functional pollinator groups

Classification of the 19 species with known pollinators (Table 1) into six syndromes (‘buzz‐bee’, ‘hummingbird’, ‘hummingbird/bat’, ‘hummingbird/rodent’, ‘flowerpiercer/rodent’ and ‘passerine; ‘six‐syndrome model’) using OOB data led to an overall median error rate of 31% over all 100 RFs. RFs were unable to separate nectar‐rewarding species correctly into separate syndromes as reflected by high levels of misclassification (‘hummingbird’, 100%; ‘hummingbird/bat’, 25%; ‘hummingbird/rodent’, 100%; ‘flowerpiercer/rodent’, 100%); classification was correct in the ‘buzz‐bee’ and ‘passerine’ (both 0% misclassification) pollinated species. However, classification of the 19 species into three syndromes (‘buzz‐bee’, ‘mixed‐vertebrate’ and ‘passerine’) noticeably reduced the overall median error rate to 5.2%. All nectar‐secreting species were correctly classified as ‘mixed‐vertebrate’ (0% misclassification). Accordingly, the ‘three‐syndrome model’ was chosen for further analyses.

### Floral characters differentiating pollination syndromes

The 20 most important floral characters differentiating the 19 Merianieae species with known pollinators into ‘buzz‐bee’, ‘mixed‐vertebrate’ or ‘passerine’ pollination syndromes are listed in Table 2 (for a complete list of all 61 characters over 100 RFs, see Fig. S2). Four characters (mode of pollen expulsion, reward type, relative position of stigma vs corolla opening, presence of filament ruptures) were particularly informative, as the removal of any of these characters reduced the mean model accuracy (and hence the accuracy of pollination syndrome classification) by > 5% (Table 2). Floral characters vary in their predictive power among syndromes: certain characters were more predictive for one syndrome than for the other two, reflected by differences in reduction in syndrome‐specific model accuracy (Table 2). For instance, all flowers in the ‘mixed‐vertebrate’ syndrome are pendant, whereas flower orientation varies in the other two syndromes. When comparing the relative importance of ‘classical’ pollination syndrome traits, eight of 14 fell within the 20 most important characters, whereas the remaining six were of less importance (Table 2). The latter include colour, scent, symmetry, corolla diameter and inflorescence position (Table S6).

### Pollination syndromes in Merianieae

Pollination syndromes and pollinator behaviour (observed by ASD, Table 1) are described on the basis of species with known pollinators; a syndrome summary is provided in Table 3 and a more detailed description is given in Notes S2.

**Table 3:** Summary of floral characters characterizing the three pollination syndromes (‘bee’, ‘mixed‐vertebrate’ and ‘passerine’) in Merianieae and traditional pollination syndrome characters; three groups can be distinguished in the ‘buzz‐bee’ syndrome (see Fig. 3)

Floral trait	‘Buzz‐bee’	‘Mixed‐vertebrate’	‘Passerine’
Orientation of flower	Upright, horizontal	Pendant	Upright, horizontal, pendant
Corolla shape	Flat to reflexed (groups 1, 3); urceolate (group 2)	Pseudo‐campanulate	Urceolate
Corolla colour	White (groups 1, 2); lilac, orange, fuchsia	White, salmon, light pink, red	Red, light pink
Petal epidermis and gloss	Conical, matt	Flat, glossy	Flat to conical, matt
Scent	Scentless, flowery	Scentless, flowery, solvent‐like	Scentless
Reward type	Pollen	Nectar	Food bodies
Pollen expulsion mechanism	Buzzing	Salt‐shaker	Bellows
Stamen appendage shape	Small acuminate (group 1), acuminate bifid (group 2), large pyramidal (group 3)	Reduced in size, crown‐like	Bulbous
Anther reflexion	Yes (group 1), no (groups 2, 3)	Yes	No
Thecal attachment	Ventral	Lateral or ventral	Ventral
Structure of adaxial thecal wall	Corrugated, sturdy	Crumpled, soft	Smooth, sturdy
Location of pore	Ventral (group 1), dorsal (groups 2, 3)	Mostly apical	Dorsal
Relative position of stigma vs corolla opening	Far exserted	At level of corolla opening	Slightly exserted

**Figure 1:**
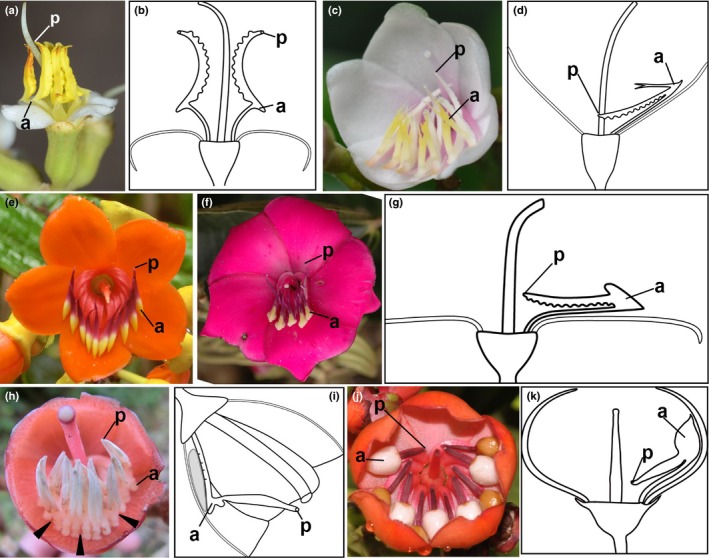
Flowers of Merianieae species. (a) Buzz‐bee‐pollinated *Graffenrieda maklenkensis*. (b) Schematic drawing of buzz‐bee‐pollinated *Graffenrieda* with reflexed corolla and radially symmetric androecium with erect stamens; note corrugated thecal wall. (c) Buzz‐bee‐pollinated *Adelobotrys adscendens*. (d) Schematic drawing of buzz‐bee‐pollinated *Adelobotrys* with urceolate corolla and heterantherous, monosymmetric androecium with geniculate stamens; note corrugated thecal wall. (e) Buzz‐bee‐pollinated *Meriania hernandoi* with reflexed corolla and isomorphic geniculate stamens. (f) Buzz‐bee‐pollinated *M. maxima* with reflexed corolla and heteranthery. (g) Schematic drawing of ‘buzz‐bee’ syndrome *Meriania* flower with reflexed corolla and monosymmetric androecium with geniculate stamens; note corrugated thecal wall. (h) Hummingbird/bat‐pollinated *M. tomentosa* with pseudo‐campanulate corolla and reflexed stamens; arrowheads indicate site of nectar aggregation. (i) Schematic drawing of ‘mixed‐vertebrate’ flower with pseudo‐campanulate corolla and monosymmetric androecium with erect stamens; grey‐shaded area indicates nectar aggregation between stamens and corolla. (j) Passerine‐pollinated *Axinaea costaricensis*. (k) Schematic drawing of ‘passerine’ syndrome flower with urceolate corolla and monosymmetric androecium with bulbous stamen appendages serving as food bodies for passerines. a, Appendage of one stamen; p, pore of one stamen.

**Figure 2:**
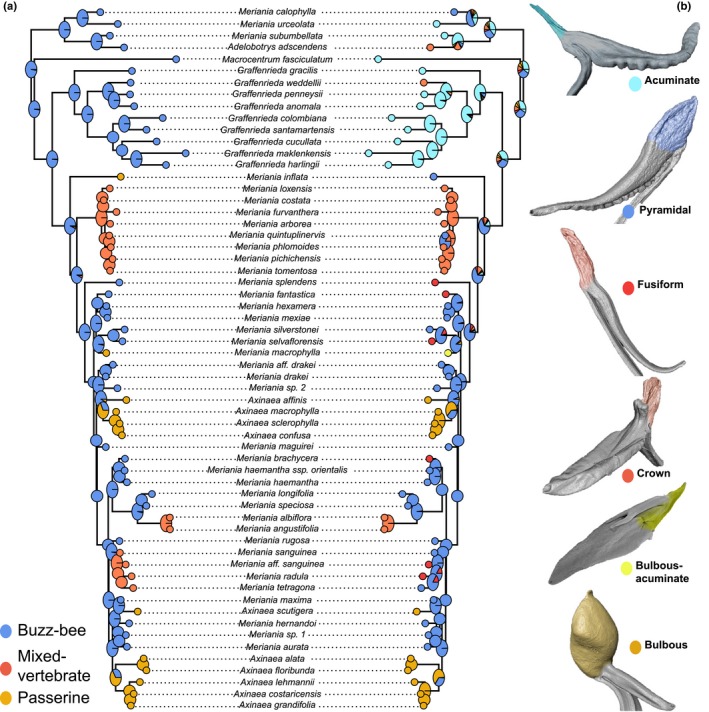
Stochastic character mapping of the three pollination syndromes (‘buzz‐bee’, ‘mixed‐vertebrate’ and ‘passerine’) and stamen appendage evolution in Merianieae. Circles at the nodes represent ancestral states estimated from 1000 mapping runs using the ‘equal rates’ (‘ER’) model. (a) The ‘buzz‐bee’ syndrome represents the ancestral pollination system in Merianieae and repeated independent shifts occurred to the ‘mixed‐vertebrate’ and the ‘passerine’ syndrome. (b) Evolution of the primary stamen appendage, with the largest diversity of primary appendage types (acuminate, pyramidal, fusiform) found within the ‘buzz‐bee’ syndrome, two types (crown and fusiform) found within the ‘mixed‐vertebrate’ syndrome and bulbous appendages (bellows organs) restricted to the ‘passerine’ syndrome. Single stamens from computed tomography (CT) scans and scanning electron microscopy (SEM) are shown; primary appendages are coloured, secondary appendages (if present) were not considered (*Graffenrieda weddellii*, acuminate; *Meriania hernandoi*, pyramidal; *M. fantastica*, fusiform; *M. phlomoides*, crown; *M. macrophylla,* bulbous‐acuminate; *Axinaea costaricensis*, bulbous).

Within the ‘buzz‐bee’ syndrome, three major groups have been distinguished (*Graffenrieda* species, group 1; *Adelobotrys adscendens*, group 2; *Meriania* species, group 3), and syndrome description is organized accordingly. Features shared by all ‘buzz‐bee’ syndrome species in Merianieae are the pollen reward and buzz‐pollination (Table 3). Corollas are wide bowl‐shaped to reflexed with papillate petal epidermis, providing a landing platform for pollinating bees (Figs 1a–g, S3a). Flower colours range from white to orange, fuchsia and lilac, with stamens forming a strong colour contrast against the petals (Fig. 1a,c,e,f). Stamens are either distributed more or less regularly in the flower (Fig. 1a,b, group 1) or arranged on one side of the flower (thus monosymmetric appearance, Fig. 1c–g, groups 2 and 3); heteranthery is found in some species in groups 2 and 3. Anthers can be erect (group 1), bringing pores close to the stigma (Fig. 1b), or remain geniculate (the condition found in bud stage in all species) with pores remaining close to the base of the style in the floral centre (Fig. 1d,g; groups 2 and 3). Stamen appendages in Merianieae are always dorsal; in groups 2 and 3 conspicuous and large (Figs 1e,f, 2b), in group 1 small and acuminate (Fig. 1c). Thecae are located on the ventral side of the connective and usually have strongly corrugated and rigid walls (Figs 1b,d,g, S3d,g); pollen can only be released by applying strong vibrations (buzzes). Pores may be located on the ventral (group 1) or dorsal (groups 2 and 3) side of the anther. Styles are usually exserted and often strongly curved right beneath the stigma. In many species, stigmas are small and punctiform. In species of groups 1 and 2 (flower diameter < 2 cm), visiting bees were seen to crouch above the entire androecium, head pointing towards the flower centre, and buzzing the entire androecium. In large‐flowered species of group 3 (flower diameter > 2 cm), pollinating bees oriented their bodies in parallel to individual stamens, with their head at the appendage and their abdomen pointing towards the pores. They bit into the appendage and buzzed individual stamens at a time. Thus, the ‘buzz‐bee’ syndrome encompasses three distinct flower morphologies and two different types of interaction between flowers and buzzing bees.

Flowers belonging to the ‘mixed‐vertebrate’ syndrome are recognized by nectar rewards secreted from stamens and pseudo‐campanulate, pendant flowers (Fig. 1h,i), with a flat petal epidermis and glossy appearance (Fig. S3b). Colours range from white, pinkish, salmon to scarlet red, and flowers are often scented. All species have androecia arranged on one side of the flower and stamens undergoing a strong deflexion movement in the early phase of anthesis, bringing pores close to stigmas (anthers erect, Fig. 1i). Stamen appendages are mostly smaller than in bee‐pollinated group 3 species (Fig. 2b), and relatively inconspicuous coloration (same colour as anther) in some species (e.g. *M. tomentosa* (hummingbird/bat)), but larger and contrasting in colour to thecae in others (e.g. *M. sanguinea* (hummingbird/rodent)). Heteranthery is absent in all species with known pollinators. In many species, thecae are attached laterally to the connective and have a soft, easily deformable (e.g. by a hummingbird's bill) wall (Fig. S3e,h). Apical anther pores are usually directed towards the stigma. Styles are often straight, not exceeding the corolla length, and often bear enlarged, slightly flattened stigmas. Vertebrate pollinators insert their bills, tongues or heads into the pseudo‐campanulate corollas to lick nectar aggregated on petals beneath the stamens. To reach the nectar, they have to push through the densely arranged anthers and thereby touch the soft, laterally attached thecae and cause pollen release from the apical pores. As all stamens are arranged with pores pointing downwards, out of the pendant flower, we term this mechanism ‘salt‐shaker’‐like pollen release. Table 3 summarizes the most important features differentiating the ‘mixed‐vertebrate’ from the ‘buzz‐bee’ syndrome: pendant, pseudo‐campanulate flowers in combination with erect stamens, nectar rewards, and soft, easily deformable thecae from which pollen can be released by applying external pressure.

The ‘passerine’ pollination syndrome is characterized by food body rewards provided by bulbous stamen appendages and urceolate corollas (Figs 1j,k, 2b) with a flat petal epidermis (Fig. S3c). Colours range from white, light pink to red. In all species, the brightly coloured stamen appendages form a strong colour contrast with the corolla. Stamens are arranged on one side of the flower (monosymmetric) and, in contrast with the ‘mixed‐vertebrate’ syndrome, they do not deflex during anthesis, so that pores remain more or less around the mid‐length of the style (Fig. 1k). Most species show moderate heteranthery (appendage volume and colour). Thecae are located on the ventral side of the connective and have a smooth, sturdy wall (Fig. S3f,i). Pores are located on the dorsal side of the anther. Styles are usually partially exserted from the urceolate corollas, with relatively small, conical stigmas. Pollen release is ultimately connected to the ubiquitous bulbous appendages: besides functioning as sugary food body reward, the bulbous appendages work as ‘bellows’ organs (Dellinger *et al*., 2014). When passerines grab the appendages for consumption, the compression forces contained air into and through the thecae, dusting the birds with pollen grains that are ejected out of the apical thecal pores. Thus, the bulbous stamen appendages are the most important character differentiating the ‘passerine’ syndrome from both ‘buzz‐bee’ and ‘mixed‐vertebrate’ syndromes (Table 3).

### Estimation of pollination syndromes and ancestral character estimation

All 42 species, for which pollinators were unknown, could be classified into one of the three pollination syndromes using RF. Group assignment over 100 RFs was 100% consistent in 41 species and 97% consistent in one species (Table S4). Estimation yielded 27 ‘buzz‐bee’ syndrome flowers in the genera *Meriania*,* Graffenrieda*,* Macrocentrum* and *Adelobotrys*, six ‘mixed‐vertebrate’ syndrome flowers in the genus *Meriania*, and nine ‘passerine’ syndrome flowers in the genera *Meriania* and *Axinaea*. Buzz‐bee pollination was resolved as the ancestral pollination system at the root with the equal rates model performing best (Akaike information criterion (AIC), 70.4; log‐likelihood, −34.2; scaled likelihood: ‘buzz‐bee’, 97.7%; ‘mixed‐vertebrate’, 1.1%; ‘passerine’, 1.1%; AIC of ‘all rates different’ model, 76.6; log‐likelihood, −32.3; see Table S7 for syndrome transition rates; likelihood ratio‐test, *P* = 0.57). The mapping of three crucial traits (‘appendage shape’ (Fig. 2b), ‘relation between stigma and corolla opening’ and ‘filament ruptures’ (Figs S4, S5)) confirmed the trait change patterns found in RF analyses.

### Disparity of different syndromes

**Figure 3:**
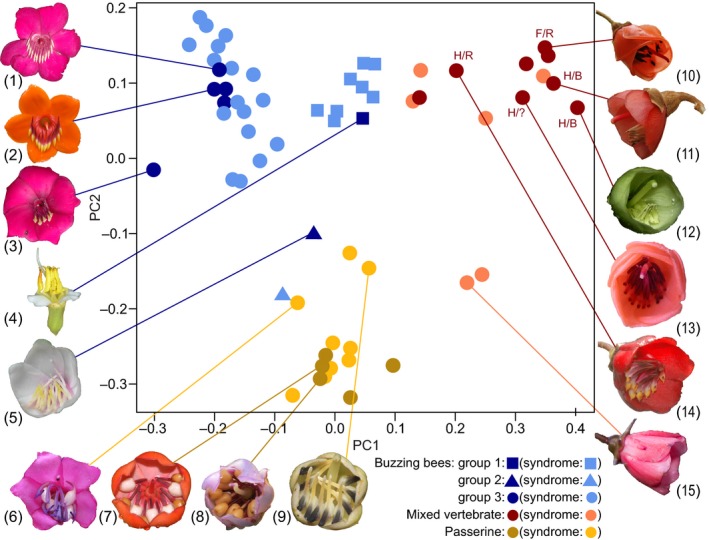
Morphospace of the three Merianieae pollination syndromes: ‘buzz‐bee’, ‘mixed‐vertebrate’ and ‘passerine’. Colours indicate known pollinators and pollination syndromes; functional pollinator groups of the ‘mixed‐vertebrate’ syndrome (H/B, hummingbird/bat; H/R, hummingbird/rodent, F/R, flowerpiercers/rodent; H/?, hummingbird/unknown) are given to underpin convergence despite pollination by different functional groups. The ‘buzz‐bee’ syndrome is scattered in three clusters (group 1 (flower 4), group 2 (flower 5), group 3). Single species were selected to exemplify the morphological diversity of the group: (1) *Meriania maguirei*, (2) *M. hernandoi*, (3) *M. maxima*, (4) *Graffenrieda maklenkensis*, (5) *Adelobotrys adscendens*, (6) *M. macrophylla*, (7) *Axinaea costaricensis*, (8) *A. sclerophylla*, (9) *M. inflata*, (10) *M. furvanthera*, (11) *M. tomentosa*, (12) *M. phlomoides*, (13) *M. costata*, (14) *M. sanguinea* and (15) *M. angustifolia*. [Correction added after online publication 12 October 2018: the figure and associated legend have been updated.]

PCoA on the 61 species showed clear grouping according to pollination syndromes and occupation of different areas of morphospace (Fig. 3); 59.2% of the variation was explained by the first three axes. Significant differences in morphospace occupation were detected between syndromes (*F* = 21.785, df = 2, *P* < 0.0001; for details on *post‐hoc* group differences, see Table S8). Also, syndromes differed significantly in disparity (Kruskal–Wallis: Chi^2^ = 65.7, df = 2, *P* < 0.0001; for details on *post‐hoc* group differences, see Table S9). The ‘buzz‐bee’ pollination syndrome was morphologically most diverse (mean pairwise dissimilarity $\overset{¯}{D}$ = 0.364 ± 0.131 (SD)), i.e. occupied the largest area in the morphospace. Three ‘buzz‐bee’ syndrome clusters could be distinguished, encompassing very different floral morphologies: small‐flowered species with reflexed petals and erect stamens (Fig. 1a,b; group 1, differentiated mostly by PCO3, Fig. S6); large‐flowered species with reflexed petals and geniculate stamens (Fig. 1e–g; group 3), which occupied a large and distinct area of the space (negative PCO1, positive PCO2); and bee‐pollinated species with urceolate corollas and slightly erect stamens (Fig. 1c,d, group 2), which occupied an area close to the ‘passerine’ syndrome. The second largest disparity was found in the ‘mixed‐vertebrate’ syndrome ($\overset{¯}{D}$ = 0.318 ± 0.130), which is clearly differentiated from the ‘bee’ syndrome by PCO1 and from the passerine syndrome by PCO2. The different functional pollinator groups (‘hummingbird’, ‘hummingbird/bat’, ‘hummingbird/rodent’, ‘flowerpiercer/rodent’) could not be distinguished in the morphospace (Fig. 3). The ‘passerine’ syndrome occupied the smallest area ($\overset{¯}{D}$ = 0.242 ± 0.087) of the space, differentiated by PCO2. When assessing the contribution to total disparity, the ‘buzz‐bee’ syndrome alone contributed 51.3%, whereas the ‘mixed‐vertebrate’ and ‘passerine’ syndromes only contributed 28.8% and 20.0%, respectively.

## Discussion

Our results corroborate the general concept of pollination syndromes and allow the detection and description of convergence of multiple floral traits into three distinct pollination syndromes in Merianieae: the ancestral ‘buzz‐bee’, the ‘mixed‐vertebrate’ and the ‘passerine’ syndromes (Fig. 2a). These syndromes are best described by a series of traits specific to Merianieae, rather than by ‘classical’ pollination syndrome characters, as indicated by the relatively low contribution to the differentiation model of the latter type of character (Tables 2, S6; Faegri & van der Pijl, 1979; Ollerton *et al*., 2009; Serrano‐Serrano *et al*., 2017). Our results generally support the hypothesis that ‘attraction’ traits (e.g. exposure of flower, display size, scent, colour, flower symmetry and timing of anthesis) are less important in differentiating syndromes than ‘efficiency’ traits involved in the direct physical interaction between flower and pollinators (e.g. flower shape and orientation, position of reproductive organs). This is particularly important for two reasons. First, most studies on phenotypic selection detected selection only on attraction traits (e.g. Armbruster *et al*., 2005). Attraction traits, however, can be subject to opposing selection in the presence of floral antagonists or trade‐offs in pollen delivery, and hence selection will be less consistent and weaker than on traits involved in accurate pollen transfer (e.g. Armbruster *et al*., 2005; Strauss & Whittall, 2006; Rosas‐Guerrero *et al*., 2011). Second, ‘classical’ syndrome characters, such as the ‘attraction’ traits colour and display size, are regularly included in studies on pollination syndromes (Lagomarsino *et al*., 2017; Wilson *et al*., 2017), whereas ‘efficiency’ traits, such as anther–stigma distance, have generally received less attention. At least in Merianieae, certain ‘classical’ syndrome characters either did not vary consistently between syndromes (e.g. timing of anthesis (most flowers are open during day‐ and night‐time); flower size (both smallest and largest flowers are found in the ‘buzz‐bee’ syndrome)), or they contradicted traditional syndrome expectations (e.g. floral colour (many pale pink and white bird‐pollinated flowers instead of the ‘red–bird’ association)). We wish to point out, however, that one ‘classical’ syndrome trait (reward type) involved in pollinator attraction was the second‐most important character in differentiating syndromes (see discussion on association of reward and androecium).

The difficulty in delimiting ‘classical’ pollination syndromes in Merianieae is further illustrated by the ‘mixed‐vertebrate’ syndrome. Pollination syndrome theory (e.g. Faegri & van der Pijl, 1979) would split the various combinations of different vertebrate pollinators that we observed visiting Merianieae species (‘hummingbird/?’, ‘hummingbird/bat’, ‘hummingbird/rodent’ and ‘flowerpiercer/rodent’) into separate functional groups (hummingbirds, flowerpiercers, bats and rodents) based on differences in timing of activity (diurnal/nocturnal), means of localizing flowers (visual/scent/echolocation), foraging behaviour (hovering/perching), morphological fit with flowers (tubular/bowl‐shaped flowers) and nectar preferences (sucroses/hexoses). However, our RF and disparity analyses did not support syndromes related to any individual pollinator group or did not separate syndromes related to the different mixed pollinator assemblages. On the contrary, our results underscore that these pollinator groups are part of the same ‘functional group’ based on their shared interest in the nectar reward and their ability to cause pollen release via the ‘salt‐shaker’ mechanism. Indeed, the ‘mixed‐vertebrate’ syndrome in Merianieae could encompass different cases of specialized bimodal pollination systems, which are systems representing intermediate adaptations to two different (equally effective) functional pollinator groups (Manning & Goldblatt, 2005). Mixed pollinator assemblages can also be the result of retaining ancestral pollinators whilst being specialized on a more effective primary pollinator (Rosas‐Guerrero *et al*., 2014). In bird syndromes, ancestral bee pollinators are disproportionately common, as well as ancestral bird pollinators in bat syndromes (e.g. Buzato *et al*., 1994; Wilson *et al*., 2007; Tripp & Manos, 2008). In Merianieae, bees have not been observed as pollinators in either the ‘mixed‐vertebrate’ or the ‘passerine’ syndrome. The ‘mixed‐vertebrate’ syndrome, however, could potentially represent a transition stage between ancestral bird and novel bat/rodent pollination, or vice versa. Alternatively, pollinator shifts in Merianieae could have passed directly from a buzz‐bee system to the different combinations of vertebrate pollinators. A salient feature of all Merianieae with a ‘mixed‐vertebrate’ syndrome is that they all combine a diurnal with a nocturnal pollinator. We hypothesize that such a ‘24/7 access’ to pollinators may be an important adaptive advantage that could have driven these pollinator shifts in Merianieae with Andean distribution (Varassin *et al*., 2008). A few other systems employing hummingbirds and bats as pollinators are known from Neotropical cloud forests (e.g. *Aphelandra* (Acanthaceae), Muchhala *et al*., 2009; *Encholirium* (Bromeliaceae), Queiroz *et al*., 2016), and the combination of these pollinators has been interpreted as a pollination assurance mechanism under harsh montane weather conditions. However, the diversity of combinations of different functional groups in Merianieae is unparalleled in other families. More detailed studies on the population level of species belonging to the ‘mixed‐vertebrate’ syndrome may allow the testing of the hypotheses outlined above and may shed light on the evolutionary history of pollinator shifts in Merianieae.

Experimental studies show that selection, and hence pollination syndrome evolution, operates on complex trait combinations, which do not always match ‘classical’ syndromes in all traits. Instead, they may represent clade‐specific syndromes, which are possibly phylogenetically constrained (Smith & Rausher, 2008; Fenster *et al*., 2015; O'Meara *et al*., 2016; Wilson *et al*., 2017). Buzz‐bee pollination in Merianieae represents a highly specialized pollination system in itself (Buchmann, 1983). It is possible that the ancestral ‘buzz’ morphology in Merianieae, with relatively open corollas and poricidal anthers, partly prevented the evolution of the group towards derived ‘classical’ syndromes, which have not originated from buzz‐pollinated flowers. Compared with other systems, access to flowers is not physically restricted by the corolla in Merianieae (e.g. no narrow corolla tubes typical of the classical hummingbird syndrome), and nectar rewards can be retrieved by a variety of pollinators. In pollen‐rewarding Merianieae, however, poricidal anthers strictly confine access to the reward to bees capable of buzzing. Poricidal anthers were retained in all Merianieae species, which could be due to an anatomical constraint (lack of endothecium) hindering the evolution of longitudinal anther dehiscence (Keijzer, 1987). Interestingly, in the Melastomataceae genus *Miconia*, this constraint was apparently overcome as longitudinal anther dehiscence has evolved at least three times (Goldenberg *et al*., 2003) and has resulted in pollination by non‐buzzing insects (Brito *et al*., 2017). Conserving the poricidal anther morphology whilst shifting to non‐buzzing pollinators in Merianieae, however, made the evolution of alternative pollen expulsion mechanisms a necessity. It is thus not surprising that the pollen expulsion mechanism was the most important floral trait separating the three pollination syndromes in Merianieae, with buzzing in the ‘buzz‐bee’ syndrome, the ‘salt‐shaker’ mechanism in the ‘mixed‐vertebrate’ syndrome and the ‘bellows’ mechanism in the ‘passerine’ syndrome. The complex functioning of these two new mechanisms was achieved by considerable morphological modifications in the androecium (Fig. 2b). In the ‘mixed‐vertebrate’ syndrome, stamens have deflexed so that pores point towards the opening of the pendant corolla, the location of the thecae has changed from dorsal to lateral, and thecal walls have softened in most species so that pollen is easily released when external pressure is applied (e.g. by a hummingbird's bill). Together, these changes promote the ‘salt‐shaker’‐like release of pollen. In the ‘passerine’ syndrome, stamen appendages have been modified into inflated bulbous ‘bellows’ organs which cause pollen ejection from thecae when seized by the foraging passerines (Dellinger *et al*., 2014).

In addition to promoting pollen dispersal, the androecium provides the reward in all three syndromes: pollen in the ‘buzz‐bee’ syndrome, nectar in the ‘mixed‐vertebrate’ syndrome, which is secreted from staminal filament ruptures, and sucrose‐rich food bodies in the ‘passerine’ syndrome, which are formed by the bulbous stamen appendages (Dellinger *et al*., 2014). This androecium–reward association in Merianieae is particularly important when compared with rewarding structures across angiosperms: both staminal food bodies and nectar release by stamens are otherwise rare. Staminal food bodies are mainly associated with beetle pollination (e.g. Cyclanthaceae, Bernhard, 1996; Calycanthaceae, Gottsberger, 2015) and staminal nectar release usually occurs by specialized nectaries at the filament base, but not by ruptures along filaments as in Merianieae (staminal nectar release has been reported in Laurales, Magnoliales, Caryophyllales and Geraniales; Bernardello, 2007). In addition to the pollen transfer and rewarding function of the androecium, stamen appendages in buzz‐bee‐pollinated species form strong colour contrasts with the corolla and therefore also carry an advertisement function. This function has been retained in the ‘passerine’ syndrome, where bulbous appendages also contrast against petals, and partially in the ‘mixed‐vertebrate’ syndrome (in some species (Fig. 3, flower 14), appendages form the contrast; in others, entire stamens (Fig. 3, flowers 10, 11 and 13) or there is no contrast (Fig. 3, flower 12)). Thus, the androecial multifunctionality of the buzz syndrome has been almost completely retained throughout pollinator shifts in Merianieae and both the complex pollen expulsion mechanisms and unusual rewarding structures are the result of the evolutionary starting point (buzz‐pollination syndrome). The strong effect of such evolutionary starting points (genetic context) on adaptation (evolutionary outcome) as a source of trait diversity was recognized by Darwin (Darwin, 1859; Armbruster, 2002).

Merianieae pollination syndromes differed markedly in their levels of floral disparity, with the ‘buzz‐bee’ syndrome clearly being most variable, occupying three distinct areas of morphospace. This is in line with previous studies describing buzz‐pollinated Melastomataceae as ‘wandering on an adaptive peak’ (Macior, 1971; Reginato & Michelangeli, 2016). Apparently, the evolutionarily successful buzz‐pollination system does not strictly constrain the floral phenotype, but can be achieved by a variety of floral constructions, united by a common reward type (pollen) and pollen expulsion mechanism (buzzing). This, in turn, broadens the exploitable buzz‐bee pollinator niche. A typical buzz syndrome flower is often associated with the architecture of the ‘*Solanum*‐type’ flower (Buchmann, 1983; de Luca & Vallejo‐Marín, 2013), a small, polysymmetric, pendant flower with reflexed petals and anthers forming a cone on which the bees crouch for buzzing. In the Merianieae species studied here, this phenotype is only realized by a part of the species (buzz‐bee group 1, Fig. 3, flower 4). All other buzz‐pollinated Merianieae have relatively large flowers with a polysymmetric perianth, but a distinctly monosymmetric androecium. Similar buzz‐pollinated flowers are present in the genus *Senna* (Fabaceae, Marazzi & Endress, 2008; Amorim *et al*., 2017). Although *Senna* flowers are usually urceolate with pronounced heteranthery (Buchmann, 1983; Marazzi *et al*., 2007), this character combination is found only in buzz‐bee group 2 (Fig. 3, flower 5). In comparison with the ‘buzz‐bee’ syndrome, the ‘mixed‐vertebrate’ and ‘passerine’ syndromes show much lower levels of disparity. Apparently, migration from the ‘buzz‐bee plateau’ happened along two relatively narrow ridges in combination with a change in reward type, pollen expulsion mechanism, corolla shape and androecial arrangement. Although not yet formally tested, this seems to be in line with pollinator shifts reported for the three other Neotropical Melastomataceae tribes (Blakeeae, Melastomateae, Miconieae, e.g. Goldenberg *et al*., 2008; Varassin *et al*., 2008; Penneys & Judd, 2011). As in Merianieae, the vast majority of species in the rest of Melastomataceae are buzz‐bee‐pollinated (*c*. 89%, Renner, 1989) and show a tremendous diversity of floral morphologies. Shifts to alternative specialized and more generalized pollination systems always involve changes in reward type and pollen release (Renner, 1989; Varassin *et al*., 2008; Brito *et al*., 2016).

In conclusion, our results provide an important step forward in the study of floral morphological and functional adaptations to different pollinator groups. We demonstrate that the highly specialized buzz‐pollination syndrome largely channelled the evolution of alternative pollination systems, and that the multi‐functionality of the androecium (pollen expulsion, reward, attraction) was retained throughout pollinator shifts. Our results further emphasize the value and validity of the pollination syndrome concept, but, at the same time, point out that pollination syndromes need to be evaluated carefully in each study group.

## Author contributions

ASD and JS conceived the idea and designed the study, ASD, DF‐F, DSP, MA, FA and FAM carried out fieldwork and flower sampling, YS assisted in HR‐XCT scanning, MC gave support in statistical analyses and WSA in discussions on pollination concepts. All authors contributed to writing and revising the manuscript.

## Supporting information

Please note: Wiley Blackwell are not responsible for the content or functionality of any Supporting Information supplied by the authors. Any queries (other than missing material) should be directed to the *New Phytologist* Central Office.

**Fig. S1** Nectar‐producing *Meriania* species with known pollinators grouped into the ‘mixed‐vertebrate’ pollination syndrome.
**Fig. S2** Ranking of all 61 floral traits by decrease in Gini index using random forest (RF) analyses.
**Fig. S3** Structural properties of petals and stamens in Merianieae.
**Fig. S4** Stochastic character mapping of pollination syndromes and the ‘filament structure’.
**Fig. S5** Stochastic character mapping of pollination syndromes and the character ‘relation style to corolla’.
**Fig. S6** Merianieae morphospace PC1–3.
**Notes S1** Sixty‐one floral characters and character states recorded for Merianieae.
**Notes S2** Detailed description of Merianieae pollination syndromes.
**Table S1** Merianieae species included in the morphospace and information on sampling localities.
**Table S2** Pollinator information for the 19 Merianieae species used for the delimitation of pollination syndromes.
**Table S3** Misclassification percentage of 19 Merianieae species with known pollinators.
**Table S4** Probability of pollinator classification by random forest (RF) analyses.
**Table S5** Merianieae species included in the full phylogeny, sampling localities, collector and voucher information and GenBank accession numbers for genes used for construction of the phylogeny.
**Table S6** Predictive value of floral characters used in traditional pollination syndromes.
**Table S7** Estimated average number of pollination syndrome shifts across 1000 stochastic character mappings.
**Table S8** Results from *post‐hoc* test on morphological differences between pollination syndromes.
**Table S9** Results from *post‐hoc* test on significant differences in disparity between pollination syndromes.
