# Supplementary material for: Beyond buzz‐pollination – departures from an adaptive plateau lead to new pollination syndromes
Source: New Phytol. 2018 Oct 12;221(2):1136–49. doi: 10.1111/nph.15468 (PMC6492237; doi:10.1111/nph.15468)
Supplement: Supplementary file 1 — Fig. S1 Nectar‐producing Meriania species with known pollinators grouped into the ‘mixed‐vertebrate’ pollination syndrome. Fig. S2 Ranking of all 61 floral traits by decrease in Gini index using random forest (RF) analyses. Fig. S3 Structural properties of petals and stamens in Merianieae. Fig. S4 Stochastic character mapping of pollination syndromes and the ‘filament structure’. Fig. S5 Stochastic character mapping of pollination syndromes and the character ‘relation style to corolla’. Fig. S6 Merianieae morphospace PC1–3. Notes S1 Sixty‐one floral characters and character states recorded for Merianieae. Notes S2 Detailed description of Merianieae pollination syndromes. Table S1 Merianieae species included in the morphospace and information on sampling localities. Table S2 Pollinator information for the 19 Merianieae species used for the delimitation of pollination syndromes. Table S3 Misclassification percentage of 19 Merianieae species with known pollinators. Table S4 Probability of pollinator classification by random forest (RF) analyses. Table S5 Merianieae species included in the full phylogeny, sampling localities, collector and voucher information and GenBank accession numbers for genes used for construction of the phylogeny. Table S6 Predictive value of floral characters used in traditional pollination syndromes. Table S7 Estimated average number of pollination syndrome shifts across 1000 stochastic character mappings. Table S8 Results from post‐hoc test on morphological differences between pollination syndromes. Table S9 Results from post‐hoc test on significant differences in disparity between pollination syndromes. [file NPH-221-1136-s001.pdf]

***New Phytologist* Supporting Information**

Article title: **Beyond buzz-pollination – departures from an adaptive plateau lead to new pollination syndromes**

Authors: Agnes S. Dellinger, Marion Chartier, Diana Fernández-Fernández, Darin S. Penneys, Marcela Alvear, Frank Almeda, Fabián A. Michelangeli, Yannick Staedler, W. Scott Armbruster, Jürg Schönenberger

Article acceptance date: 1 August 2018.

The following Supporting Information is available for this article:

Table S1. Merianieae species included in morphospace and information on sampling localities.
Table S2. Pollinator information for the 19 Merianieae species used for delimiting pollination syndromes.
Table S3. Misclassification percentage of 19 Merianieae species with known pollinators.
Table S4. Probability of pollinator classification by Random Forest Analyses (RF**).**
Table S5. Merianieae species included in the full phylogeny, sampling localities, collector and voucher information and GenBank accession numbers for genes used for constructing the phylogeny.
Table S6. Predictive value of floral characters used in traditional pollination syndromes.
Table S7. Estimated average number of pollination syndrome shifts across 1000 stochastic character mappings.
Table S8. Results from post-hoc test on morphological differences between pollination syndromes.
Table S9. Results from post-hoc test on significant differences in disparity between pollination syndromes.
Figure S1. Nectar producing *Merania* species with known pollinators grouped into the ‘mixed vertebrate’ pollination syndrome.
Figure S2. Ranking of all 61 floral traits by decrease in Gini Index using RF analyses.
Figure S3. Structural properties of petals and stamens in Merianieae.
Figure S4. Stochastic character mapping of pollination syndromes (left) and the ‘filament structure’ (right).
Figure S5. Stochastic character mapping of pollination syndromes (left) and the character ‘relation style to corolla’ (right).
Figure S6. Merianieae morphospace PC1-3.
Notes S1. 61 floral characters and character states recorded for Merianieae.
Notes S2. Detailed description of Merianieae pollination syndromes.

Table S1. Merianieae species included in morphospace and information on sampling localities.

species	collectin no	collector	country	state/province	elevation	collection date	voucher
Adelobotrys adscendens	FA10230	Frank Alemda	Colombia	Valle del Cauca	593	04.02.2011	CAS 1120080
Axinaea affinis	AD41	Agnes Dellinger	Ecuador	Azuay	3200	29.11.2012	-
Axinaea alata	NM55309	M Nee	Bolivia	Cochabamba	2845	03.05.2007	NY02424039
Axinaea floribunda	FM1981	Fabián Michelangeli	Peru	Cusco	2558	21.06.2012	NY02540381
Axinaea confusa	AD127	Agnes Dellinger	Ecuador	Loja	1800	13.09.2016	WU 0092828
Axinaea costaricensis	AD75	Agnes Dellinger	Costa Rica	San José	2600	03.02.2016	WU
Axinaea grandifolia 1	MA1697	Marcela Alvear	Colombia	Narino	2922	25.01.2013	CAS 1156779
Axinaea grandifolia 2	FM650	Fabián Michelangeli	Venezuela	Merida	2500-2700	13.01.2001	BH
Axinaea lehmannii	FA10322	Frank Alemda	Colombia	Valle del Cauca	2080	13.02.2011	CAS
Axinaea macrophylla	DSP1598	Darin S. Penneys	Ecuador	Morona-Santiago	2400	28.09.2003	NY02450495
Axinaea sclerophylla	AD24	Agnes Dellinger	Ecuador	Loja	2750	20.10.2012	WU 0072429
Axinaea scutigera	AD129	Agnes Dellinger	Ecuador	Napo	2715	14.10.2016	WU 0092827
Graffenrieda anomala	FA10434	Frank Alemda	Colombia	Chocó	99	31.01.2012	CAS 1127619
Graffenrieda colombiana	MA1862	Marcela Alvear	Colombia	Putumayo	699	18.02.2013	CAS 1156711
Graffenrieda cucullata	MA1735	Marcela Alvear	Colombia	Narino	1362	02.02.2013	CAS 1156955
Graffenrieda gracilis	FM1763	Fabián Michelangeli	Peru	Amazonas	764	18.03.2012	NY02540393
Graffenrieda harlingii	CU1843	Carmen Ulloa	Ecuador	Loja	2465-3230	04.06.2010	NY1596631
Graffenrieda maklekensis	FA10643	Frank Alemda	Colombia	Santander	1900	09.03.2012	CAS 1127617
Graffenrieda penneysii	AD184	Agnes Dellinger	Ecuador	Zamora-Chinchipe	2539	13.11.2017	QCNE
Graffenrieda santamartensis	FA10636	Frank Alemda	Colombia	Santander	1715	07.03.2012	CAS 1127621
Graffenrieda weddellii	MA1503	Marcela Alvear	Colombia	Risaralda	1374	05.01.2013	CAS 1155724
Macrocentrum fasciculatum	FM2144	Fabián Michelangeli	Suriname	Sipaliwini	720	20.08.2013	NYBG1637020

Meriania aff sanguinea	AD176	Agnes Dellinger	Ecuador	Carchi	3100	01.11.2017	QCNE
Meriania aff. drakei	AD141/DF2278	Agnes Dellinger	Ecuador	Pastaza	1843	13.11.2016	QCNE
Meriania albiflora	FM2211	Fabián Michelangeli	Cuba	Granma	885	08.11.2013	NYBG2361494
Meriania angustifolia	FM2241	Fabián Michelangeli	Cuba	Holguín	250	13.11.2013	NYBG02499331
Meriania arborea	FA10564	Frank Alemda	Colombia	Norte de Santander	2300	28.02.2012	CAS 1128115
Meriania aurata	AD145/DF2282	Agnes Dellinger	Ecuador	Pastaza	2208	13.11.2018	QCNE
Meriania brachycera	FA10547	Frank Alemda	Colombia	Norte de Santander	2600	26.02.2012	CAS 1127903
Meriania calophylla	FA1609	Fabián Michelangeli	Brazil	Espírito Santo	837	08.02.2011	NY1654154
Meriania cf costata	AD106/DF2214	Agnes Dellinger	Ecuador	Loja	2900	10.09.2016	WU 0092833
Meriania drakei	AD132	Agnes Dellinger	Ecuador	Napo	2052	14.10.2016	WU 0092805
Meriania sp. nov2	AD146/DF2285	Agnes Dellinger	Ecuador	Pastaza	1568	14.11.2016	WU 0092844
Meriania fantastica	MA1951	Marcela Alvear	Colombia	Putumayo	2314	16.02.2013	CAS 1156637
Meriania furvanthera	AD113/DF2236	Agnes Dellinger	Ecuador	Loja	2800	13.09.2016	WU 0092838
Meriania haemantha ssp. haemantha	FA10569	Frank Alemda	Colombia	Norte de Santander	2550	28.02.2012	CAS 1127905
Meriania haemantha ssp. orientalis	FA10651	Frank Alemda	Colombia	Santander	1700	11.03.2012	CAS 1128063
Meriania hernandoi	MA1856	Marcela Alvear	Colombia	Putumayo	2151	16.02.2013	CAS 1156636
Meriania hexamera	MA1854	Marcela Alvear	Colombia	Putumayo	2314	16.02.2013	CAS 1156638
Meriania inflata	RG2078	Renato Goldenberg	Brazil	Bahia	675	13.10.2014	NY02286571
Meriania longifolia	FA10536	Frank Alemda	Colombia	Norte de Santander	1259	25.02.2012	CAS 1127902
Meriania loxensis	AD115/DF2226	Agnes Dellinger	Ecuador	Loja	2700	12.09.2016	WU 0092836
Meriania macrophylla	MA1496	Marcela Alvear	Colombia	Risaralda	1338	05.01.2013	CAS 1156342
Meriania maguirei	AD110/DF	Agnes Dellinger	Ecuador	Loja	2850	11.09.2016	QCNE
Meriania maxima	MA1768	Marcela Alvear	Colombia	Narino	1888	06.02.2013	CAS 1155921

Meriania mexiae	MA1853	Marcela Alvear	Colombia	Putumayo	2314	16.02.2013	CAS 1156500
Meriania phlomoides	MA1733	Marcela Alvear	Colombia	Narino	1414	02.02.2013	CAS 1156124
Meriania pichinchensis	DSP1905	Darin Penneys	Ecuador	Pichincha	1930	06/12/2005	NY02500177
Meriania quintuplinervis	FA10306	Frank Alemda	Colombia	Valle del Cauca	2140	11.02.2011	CAS 1120552
Meriania radula	AD201/DF	Agnes Dellinger	Ecuador	Zamora-Chinchipe	3180	15.11.2017	QCNE
Meriania rugosa	FM1725	Fabián Michelangeli	Peru	Amazonas	2400	12.03.2012	NY02540643
Meriania sanguinea	AD108/DF2215	Agnes Dellinger	Ecuador	Loja	2850	10.09.2016	WU 0092832
Meriania selvaflorensis	MA1465	Marcela Alvear	Colombia	Caldas	1732	02.03.2011	CAS 1119760
Meriania silverstonei	FA10210	Frank Alemda	Colombia	Valle del Cauca	1960	01.02.2011	CAS 1120063
Meriania sp. nov1	AD158/DF2304	Agnes Dellinger	Ecuador	Pastaza	2533	15.11.2016	WU 0092856
Meriania speciosa	FA10219	Frank Alemda	Colombia	Valle del Cauca	1875	02.02.2011	CAS 1119942
Meriania splendens	MA1690	Marcela Alvear	Colombia	Narino	2922	25.01.2013	CAS 1156411
Meriania subumbellata	FM819	Fabián Michelangeli	Venezuela	Aragua	1550	03.01.2002	NYBG01101015
Meriania tetragona	AD187/DF	Agnes Dellinger	Ecuador	Zamora-Chinchipe	1859	14.11.2017	QCNE
Meriania tomentosa	AD105	Agnes Dellinger	Ecuador	Pichincha	1700	08.09.2016	WU 0092814
Meriania urceolata	KR1446	Karen Redden	Guyana	Cuyuni-Mazaruni	490	8.12.2002	NY02513392

Table S2. Pollinator information for the 19 Merianieae species used for delimiting pollination syndromes and as training set for Random Forest classification for pollinator estimation. The total number of days/nights when pollinator monitoring was made is given as well as the total number of hours of reviewed video material; a minimum of three 30 minute intervals was reviewed from every observation day.

species	pollinator group	source	study site	number of days filmed	number of nights filmed	hours reviewed daytime	hours reviewed nighttime
Adelobotrys adscendens	buzz-bee	A. S.Dellinger, pers. obs.	Costa Rica, Field Station La Gamba	7	-	13	-
Graffenrieda cucullata	buzz-bee	A. S.Dellinger, pers. obs.	Ecuador, Field Station Reserva Drákula	-	-	2h direct observation	-
Meriania drakei	buzz-bee	A. S.Dellinger, pers. obs.	Ecuador, Orchid Garden in Cosanga	-	-	2h direct observation	-
Meriania hernandoi	buzz-bee	A. S.Dellinger, pers. obs.	Ecuador, Orchid Garden in Cosanga	5	-	22	-
Meriania longifolia	buzz-bee	Renner 1989	-	-	-	-	-
Meriania maguirei	buzz-bee	A. S.Dellinger, pers. obs.	Ecuador, Podocarpus National Park	8	-	20	-
Meriania maxima	buzz-bee	A. S.Dellinger, pers. obs.	Ecuador, Bellavista Reserve	4	-	12	-
Meriania furvanthera	flowerpiercer/rodent	A. S.Dellinger, pers. obs.	Ecuador, Podocarpus National Park	2	3	8	7
Meriania costata	hummingbird/?bat	A. S.Dellinger, pers. obs.	Ecuador, Podocarpus National Park	2	-	5	-
Meriania quintuplinervis	hummingbird/?bat	Calderón-Sáenz 2012	-	-	-	-	-
Meriania pichichensis	hummingbird/bat	Muchhala & V.-Jarrín 2004, A. S.Dellinger, pers. obs.	Ecuador, Bellavista Cloudforest Reserve	-	-	-	-
Meriania aff. sanguinea	hummingbird/bat	A. S.Dellinger, pers. obs.	Ecuador, Guanderas Reserve	5	4	13	9.4

Meriania phlomoides	hummingbird/bat	Vogel 1997, A. S.Dellinger, pers. obs.	Costa Rica, Field Station Monteverde	5	3	10	12
Meriania tomentosa	hummingbird/bat	A. S.Dellinger, pers. obs.	Ecuador, Bellavista Cloudforest Reserve	8	7	15	6
Meriania sanguinea	hummingbird/rodent	A. S.Dellinger, pers. obs.	Ecuador, Podocarpus National Park	7	4	10	36
Axinaea confusa	passerine	Dellinger et al. 2014	-	-	-	-	-
Axinaea costaricensis	passerine	Dellinger et al. 2014	-	-	-	-	-
Axinaea macrophylla	passerine	Rojas-Nossa 2007	-	-	-	-	-
Axinaea sclerophylla	passerine	Dellinger et al. 2014	-	-	-	-	-

Table S3. Misclassification percentage of 19 Merianieae species with known pollinators when running models without the two most important predictive traits "pollen expulsion mechanism" and "reward type" (median error rate: 10.5%, 'buzz-bee': 28.6%, 'mixed-vertebrate' (MV): 0%, 'passerine': 0%). Misclassification only occurred in the two known buzz-bee pollinated species (*Adelobotrys adscendens*, *Graffenrieda cucullata*) with morphologies very distinct from the majority of buzz-bee pollinated Merianieae, which also displayed slight classification uncertainty in the full trait dataset. Classification errors disappeared when including all 61 species which encompass additional taxa sharing these distinct morphologies. Thus, models were considered accurate enough for pollination syndrome predictions.

species	known pollinator	% correct prediction
Adelobotrys adscendens	buzz-bee	0.07
Axinaea confusa	passerine	1
Axinaea costaricensis	passerine	1
Axinaea macrophylla	passerine	1
Axinaea sclerophylla	passerine	1
Graffenrieda cucullata	buzz-bee	0.01
Meriania costata	MV	1
Meriania drakei	buzz-bee	1
Meriania furvanthera	MV	1
Meriania hernandoi	buzz-bee	1
Meriania longifolia	buzz-bee	1
Meriania maguirei	buzz-bee	1
Meriania maxima	buzz-bee	1
Meriania phlomoides	MV	1
Meriania pichinchensis	MV	1
Meriania quintuplinervis	MV	1
Meriania sanguinea	MV	1
Meriania tomentosa	MV	1
Meriania aff. sanguinea	MV	1

Table S4. Probability of pollinator classification by Random Forest Analyses (RF) using 100 RFs with 500 trees each. For all species, the characters "reward type" and "pollen expulsion mechanism" were removed prior to estimation; additional characters which had to be removed due to missing data are listed in the column 'characters removed'.

species	buzz-bee	hb	pass	characters removed
Axinaea affinis	0	0	1	-
Axinaea alata	0	0	1	7, 9, 20
Axinaea cf floribunda	0	0	1	6
Axinaea grandifolia	0	0	1	22, 23
Axinaea grandifolia	0	0	1	7, 8, 9, 11, 13, 21, 46, 48, 49, 50, 51
Axinaea lehmannii	0	0	1	-
Axinaea scutigera	0	0	1	22, 23

Graffenrieda anomala	1	0	0	2, 3, 12, 13, 42, 43, 44, 45, 48, 49, 50, 51, 52
Graffenrieda colombiana	1	0	0	-
Graffenrieda gracilis	1	0	0	6, 15, 32
Graffenrieda harlingii	1	0	0	-
Graffenrieda maklekensis	1	0	0	6, 15, 32
Graffenrieda penneysii	1	0	0	32, 34, 35, 36
Graffenrieda santamartensis	1	0	0	6, 15, 32
Graffenrieda weddellii	1	0	0	-
Macrocentrum frutucosum	1	0	0	11
Meriania aff. drakei	1	0	0	-
Meriania albiflora	0	1	0	6
Meriania angustifolia	0	1	0	-
Meriania arborea	0	1	0	-
Meriania aurata	1	0	0	-
Meriania brachycera	1	0	0	-
Meriania calophylla	1	0	0	37
Meriania faldas	1	0	0	8, 9, 32
Meriania fantastica	1	0	0	-
Meriania haemantha ssp. haemantha	1	0	0	-
Meriania haemantha ssp. orientalis	1	0	0	-
Meriania hexamera	1	0	0	-
Meriania inflata	0	0	1	-
Meriania loxensis	0	1	0	-
Meriania macrophylla	0	0	1	35, 36
Meriania mexiae	1	0	0	-
Meriania radula	0	1	0	-
Meriania rugosa	1	0	0	-
Meriania selvaflorensis	1	0	0	21, 42, 43, 44, 45, 46, 48, 49, 50, 51, 52
Meriania silverstonei	1	0	0	-
Meriania sp. nov	0.97	0	0.03	-
Meriania speciosa	1	0	0	-
Meriania splendens	1	0	0	-
Meriania subumbellata	1	0	0	47
Meriania urceolata	1	0	0	6
Meriania tetragona	0	1	0	35, 36, 37

Table S5. Merianieae species included in the full phylogeny, sampling localities, collector and voucher information and Genbank accession numbers for genes used for constructing the phylogeny. "no" indicate genes where no data was obtained, 'xxxxxxxx' indicate sequences submitted to Genbank but no accession numbers received by the date of submission (28072018).

sequence_ID	ACCD	ETS	ITS	ndhf	psbk	rbcl	collected_by	Country	specimen_voucher
Adelobotrys_adscendens_FA10230_T185	MG198218	MF029158	KY991642	MF105310	MF104724	MF069642	Almeda F. 10230	Colombia	COL, CAS
Adelobotrys_barbata_T1641	KF819861	KF820580	AY460446	no	KF821781	no	Caddah M.K. 528	Brazil	UPCB
Adelobotrys_boissieriana_AF215530	no	no	no	no	no	AF215530	GENBANK ONLY	GENEBANK ONLY	GENEBANK ONLY
Adelobotrys_klugii_R&T11820	no	no	KF821398	no	no	no	GENBANK ONLY	GENEBANK ONLY	GENEBANK ONLY
Adelobotrys_macrantha_KR11159	no	no	AY966413	no	no	AF215531	Ruokolainen 11159	GENEBANK ONLY	NY
Adelobotrys_permixta_KMR1515	KF819862	MF029601	EU055643	MF105723	KF821782	MF070022	Redden K.M. 1515	Guyana	NY, US
Adelobotrys_praetexta_Schulman195	no	no	KF821399	no	no	no	GENBANK ONLY	GENEBANK ONLY	GENEBANK ONLY
Adelobotrys_ruokolainenii_Schulman219	no	no	AY966410	no	no	no	GENBANK ONLY	GENEBANK ONLY	GENEBANK ONLY
Adelobotrys_scandens_Schulman133	no	no	AY966406	AY966414	no	no	GENBANK ONLY	GENEBANK ONLY	GENEBANK ONLY
Adelobotrys_spruceana_CMK587	KF819863	KF820581	KF821400	MH760282	KF821783	MH747566	Caddah M.K. 587	Brazil	UPCB
Adelobotrys_subsessilis_T2963	no	MH781591	AY966407	MH760283	MH781651	MH747567	Michelangeli F.A. 493	Peru	BH, USM
Adelobotrys_tessmannii_KR11834	no	no	no	AY966415	no	no	GENBANK ONLY	GENEBANK ONLY	GENEBANK ONLY
Axinaea_affinis_NA	no	no	AY460447	no	no	no	Luteyn J. 14130	Ecuador	NY
Axinaea_alata_T646	KF819865	KF820583	KF821401	MH760284	KF821785	MH747568	Nee M.H. 55301	Bolivia	NY
Axinaea_confusa_AD127	no	MH781592	MH819864	MH760285	MH781652	MH747569	Dellinger A. 127	Ecuador	QCNE, W
Axinaea_costaricensis_FA10183	MG198210	MF029147	KY991632	MF105300	MF104713	MF069633	Almeda F. 10183	Colombia	COL, CAS
Axinaea_costaricensis_T365	KF819866	KF820584	KF821402	no	KF821786	no	Michelangeli F.A. 1223	Costa Rica	NY
Axinaea_fallax_T2659	MH781548	MH781593	MH819865	MH760286	MH781653	MH747570	Gonzalez M. F. 927	Colombia	COL, NY
Axinaea_floribunda_T2766	MH781549	MH781594	MH819866	no	MH781654	no	Michelangeli F.A. 1981	Peru	NY, USM
Axinaea_floribunda_T2914	MH781550	MH781595	MH819867	MH760287	MH781655	MH747571	Michelangeli F.A. 1957	Peru	NY, USM
Axinaea_grandifolia_FAM650	KF819867	KF820585	KF821404	MF105579	KF821787	MH747572	Michelangeli F.A. 650	Venezuela	BH, VEN
Axinaea_lehmannii_FA10322	MG198244	MF029184	KY991668	MF105347	MF104768	MF069679	Almeda F. 10322	Colombia	COL, CAS
Axinaea_macrophylla_cf_AD117	MH781551	no	MH819868	MH760288	MH781656	MH747573	Dellinger A. 117	Ecuador	QCNE, W
Axinaea_macrophylla_DSP1598	MG198483	no	KY991536	no	no	MF069943	Penneys D. S. 1598	Ecuador	NY

Axinaea_macrophylla_T1180	KF819870	KF820588	KF821405	no	KF821790	no	Michelangeli F.A. 1265	Venezuela	NY, VEN
Axinaea_minutiflora_T2752	no	MH781596	MH819869	no	MH781657	no	Pedraza P.P. 2203	Colombia	NY
Axinaea_nitida_T3049	MH781552	MH781597	MH819870	MH760289	MH781658	MH747574	Michelangeli F.A. 2616	Peru	NY, USM
Axinaea_pauciflora_cf_DSP1590	MG198482	no	KY991535	no	no	MF069941	Penneys D. S. 1590	Ecuador	NY
Axinaea_sclerophylla_DSP1878_T1670	MG198501	KF820586	KF821403	no	KF821788	MF069977	Ulloa C. U. 1769	Ecuador	MO
Axinaea_scutigera_MEM1758	no	no	KY991968	no	no	no	Morales, M. E. 1758	Colombia	UPTC
Axinaea_scutigera_T3337	no	no	MH819871	no	MH781659	no	Dellinger A. 129	Ecuador	QCNE, W
Axinaea_sp_T3114	no	MH781598	MH819872	no	MH781660	no	Michelangeli F.A. 2737	Peru	NY, USM
Axinaea_tomentosa_T2004	MH781553	MH781599	MH819873	MH760290	MH781661	MH747575	Michelangeli F. A. 1688	Peru	NY, USM
Axinaea_wurdackii_T3065	MH781554	MH781600	MH819874	MH760291	MH781662	MH747576	Michelangeli F.A. 2668	Peru	NY, USM
Centronia_laurifolia_DN14973	no	no	KY991530	no	MF105116	MF069925	Neill D. 14973	Ecuador	MO
Centronia_laurifolia_T3323	KF819890	no	KF821419	no	MH781663	no	Ulloa C. 1780	Ecuador	MO
Clidemia rubra	KF819953	KF820692	AY460481	AF215579	KF821892	AF215535	Michelangeli, F. A., 825 (NY)	Venezuela	NY
Eriocnema_fulva_T366_CVM222_T366_T366	KF819990	KF820735	AY460481	AY553781	KF821935	AY553777	Almeda F. 8414	Brazil	CAS
Graffenrieda_anomala_FA10434	no	MF029205	KY991689	MF105356	MF104786	MH747577	Almeda F. 10434	Colombia	COL, CAS
Graffenrieda_bella_DSP1657	MG198488	no	KY991541	MF105629	MF105149	MF069953	Penneys D. S. 1657	Panama	FLAS
Graffenrieda_colombiana_MA1862_MA2608	no	MH781601	MH819875	MH760292	MH781664	no	Alvear M. 1862	Colombia	COL, CAS
Graffenrieda_cucullata_DSP1873_T1673	MG198500	MF029543	KY991556	MF105675	KF821936	MF069976	Penneys D. S. 1873	Ecuador	NY
Graffenrieda_emarginata_cf_T3072_T3115	MH781555	MH781602	MH819876	no	MH781665	no	Michelangeli F.A. 2687	Peru	NY, USM
Graffenrieda_emarginata_DSP1890	no	MF029547	KY991559	MF105573	MF105100	MF069902	Penneys D. S. 1890	Ecuador	NY
Graffenrieda_emarginata_T1676	KF819992	KF820737	KF821476	no	KF821937	no	Ulloa C.U. 1803	Ecuador	MO
Graffenrieda_galeottii_T1936	KF819993	KF820738	AY460449	MH760293	KF821938	MH747578	David H. 3242	Colombia	HUA
Graffenrieda_glandulosa_T977	KF819994	KF820739	KF821477	MH760294	KF821939	no	Goldenberg R. 938	Brazil	UPCB
Graffenrieda_goldenbergii_T983	KF820004	MH781603	KF821485	MH760295	MH781666	MH747579	Goldenberg R. 962	Brazil	UPCB
Graffenrieda_gracilis_T975	KF819995	KF820740	KF821478	MH760296	KF821940	MH747580	Goldenberg R. 935	Brazil	UPCB
Graffenrieda_harlingii_T1671	KF819996	KF820741	KF821479	MH760297	KF821941	MH747581	Ulloa C.U. 1774	Ecuador	MO
Graffenrieda_hitchcockii_T1242	KF819997	KF820742	KF821480	no	KF821942	no	Michelangeli F.A. 359	Venezuela	BH, VEN
Graffenrieda_intermedia_T579	KF819998	KF820743	EU055684	MF105536	KF821943	MF069866	Goldenberg R. 855	Brazil	UPCB

Graffenrieda_irwinii_T2696	no	MH781604	no	MH760298	MH781667		Michelangeli F. A. 2696	Guyana	NY
Graffenrieda_jefensis_DSP1687	no	no	no	MF105633	no	MF069956	Penneys D. S. 1687	Panama	FLAS
Graffenrieda_laevicarpa_T2937	MH781556	MH781605	AY460450	MH760299	MH781668	MH747582	Goldenberg R. 1940	Brazil	UPCB
Graffenrieda_latifolia_DSP1303	KY821079	MF029485	EF683143	EU055943	MF105119	MF069928	Penneys D. S. 1303	Dominica	FLAS
Graffenrieda_latifolia_FAM794	JQ730297	KF820744	no	no	JQ730503	no	Michelangeli F.A. 794	Venezuela	BH, VEN
Graffenrieda_limbata_T786	KF819999	KF820745	KF821481	MH760300	KF821944	MH747583	Goldenberg R. 998	Brazil	UPCB
Graffenrieda_maklenkensis_T2080	no	MF029227	KY991711	MH760301	MF104805	no	Almeda F. 10643	Colombia	COL, CAS
Graffenrieda_miconioides_T773	KF820000	KF820746	KF821482	MH760302	KF821945	MH747584	Goldenberg R. 929	Brazil	UPCB
Graffenrieda_micrantha_aff_DSP1511	MG198479	MF029492	KY991532	MF105600	MF105125	MF069935	Penneys D. S. 1511	Costa Rica	FLAS
Graffenrieda_micrantha_T1373	KF820001	KF820747	KF821483	MH760303	KF821946	MH747585	Kriebel R. 5503	Costa Rica	NY
Graffenrieda_moaensis_T774	KF820002	KF820748	KF821484	MH760304	KF821947	MH747586	Goldenberg R. 931	Brazil	UPCB
Graffenrieda_moritziana_FAM832	JQ730298	KF820749	AY460451	EU055944	JQ730504	EU711390	Michelangeli F.A. 832	Venezuela	BH, VEN
Graffenrieda_penneysii_DSP1891_T2903	MH781557	MH781606	MH819877	MH760305	MH781669	MH747587	Ulloa C. 1804	Ecuador	MO
Graffenrieda_reticulata_T3028	no	MH781607	MH819878	MH760306	MH781670	MH747588	Forzza R. 7150	Brazil	RB
Graffenrieda_rotundifolia_C&R	no	no	AF215532	AF215576	no	AF215532	Genebank only	GENEBANK ONLY	GENEBANK ONLY
Graffenrieda_rufescens_T2668	MH781558	MH781608	MH819879	no?	MH781671	MH747589	Michelangeli F. A. 2214	Cuba	HAJB, NY
Graffenrieda_santamartensis_aff_FA10650	no	MF029229	KY991713	MF105371	MF104807	no	Almeda F. 10650	Colombia	COL, CAS
Graffenrieda_santamartensis_FA10193	no	MF029150	KY991634	MF105303	MF104716	MF069613	Almeda F. 10193	Colombia	COL, CAS
Graffenrieda_sessilifolia_FAM510	KF820003	KF820750	AY460452	MH760307	KF821948	MH747590	Michelangeli F.A. 510	Venezuela	BH, VEN
Graffenrieda_sp_T1028	MH781559	MH781609	no	MH760308	MH781672	MH747591	Nee M. 55646	Bolivia	NY
Graffenrieda_spnov_T3026	MH781560	no	MH819880	no	MH781673	no	Forzza R. 6590	Brazil	RB
Graffenrieda_tamana_T2286	MH781561	MF029214	KY991698	MF105359	MF104792	MH747592	Almeda F. 10540	Colombia	COL, CAS
Graffenrieda_uribei_aff_FA10222	MG198217	MF029157	KY991641	MF105309	MF104723	MF069641	Almeda F. 10222	Colombia	COL, CAS
Graffenrieda_uribei_HM17594	KF820005	KF820752	KF821486	MH760309	KF821950	MH747593	Mendoza H. 17594	Colombia	FMB
Graffenrieda_weddellii_KMR4548	MG198197	KF820753	KF821487	no	KF821951	no	Redden K.M. 4548	Guyana	NY, US
Leandra mexicana	no	KF820811	GU968799	AF215580	KF822003	AF215536	Genebank only	GENEBANK ONLY	GENEBANK ONLY
Macrocentrum_anfractum_T797	KF820085	KF820851	KF821521	MH760310	KF822037	MF070024	Redden K.M. 5676	Guyana	NY, US
Macrocentrum_brevipedicellatum_T2941	MH781562	MH781610	MH819881	MH760311	MH781674	MH747594	Radosavljevic A. 183	Guyana	NY, US

Macrocentrum_cristatum_microphyllum_T807	KF820086	no	KF821522	no?	KF822038	???????	Wurdack K.J. 4218	Guyana	NY, US
Macrocentrum_cristatum_T2943	MG198564	MH781611	KY991908	no	MF105286	MF070050	Radosavljevic A. 251	Guyana	NY, US
Macrocentrum_droseroides_T805	KF820087	KF820852	KY991906	MF105745	MF070049	MF070049	Wurdack K.J. 4188	Guyana	NY, US
Macrocentrum_fasciculatum_T969	KF820088	no	KY991909	no	KF822040	MF070051	Wurdack K.J. 4342	Guyana	NY, US
Macrocentrum_gesneriaceum_T1105	KF820089	no	KF821525	MH760312	KF822041	MH747595	Redden K.M. 5001	Guyana	NY, US
Macrocentrum_minus_T1104	KF820090	KF820854	KF821526	MH760313	KF822042	MF069618	Redden K.M. 3813	Guyana	NY, US
Macrocentrum_neblinense_DD14049	KF820091	KF820855	KF821527	MH760314	MH781675	MH747596	Daly D. 14049	Colombia	NY
Macrocentrum_parvulum_T2556	no	MH781612	MH819882	MH760315	MH781676	MH747597	Michelangeli F. A. 2158	Suriname	NY
Macrocentrum_repens_T799	KF820092	KF820856	KF821528	MF105726	KF822043	MF070025	Redden K.M. 5821	Guyana	NY, US
Macrocentrum_vestitum_T2680_T2683	no	no	MH819883	MH760316	too short	MH747598	Michelangeli F. A. 2346	Guyana	NY
Maguireanthus_ayangannae_T2770_T2789A	no	MH781613	MH819884	MH760317	MH781677	MH747599	Radosavljevic A. 325	Guyana	NY, US
Meriania_acostae_T712	MG198470	KF820875	KF821537	MH760318	KF822061	MH747600	Moran R.C. 6838	Ecuador	NY
Meriania_albiflora_T2667	MH781563	MH781614	MH819885	MH760319	MH781678	MH747601	Michelangeli F. A. 2211	Cuba	HAJB, NY
Meriania_almedae_DF61	no	no	MH819886	no	no	no	Neill D. 16923	Ecuador	MO
Meriania_amplexicaulis_DF40	no	no	MH819887	no	no	no	Fernandez D. M. 1540	Ecuador	QCN
Meriania_angustifolia_T2670	MH781564	MH781615	MH819888	MH760320	MH781679	MH747602	Michelangeli F. A. 2241	Cuba	HAJB, NY
Meriania_aracaensis_T2936	MH781565	no	MH819889	MH760321	MH781680	MH747603	Goldenberg R. 1937	Brazil	UPCB
Meriania_arborea_FA10564	MH781566	MF029219	KY991703	MF105362	MF104797	MH747604	Almeda F. 10564	Colombia	COL, CAS
Meriania_aurata_AD145	no	MH781616	MH819890	MH760322	MH781681	MH747605	Dellinger A. 145	Ecuador	QCNE, W
Meriania_barbosae_MA1459	MH781567	MF029323	KY991929	MH760323	MF104900	MF069777	Alvear M. 1459	Colombia	COL, CAS
Meriania_brachycera_FA10593	no	MF029222	KY991706	MF105365	MF104800	no	Almeda F. 10593	Colombia	COL, CAS
Meriania_brachycera_T2916	MH781568	MH781617	MH819891	MH760324	MH781682	no	Almeda F. 10531	Colombia	COL, CAS
Meriania_brevipedunculata_T2663	KJ933883	KJ933924	KJ933971	MH760325	KJ934024	no	Majure L.C. 4279	Haiti	FLAS
Meriania_calophylla_T616	KF820112	KF820876	EU055707	MF105547	KF822062	MH747606	Kollmann L. 8843	Brazil	UPCB
Meriania_calyptrata_T811	KF820113	KF820877	KF821538	no	KF822063	no	Rochelle A. 351	Brazil	USP
Meriania_compressicaulis_DSP1759	KY821078	MF029531	KY782388	MF105568	MF105097	MF069900	Penneys D. S. 1759	Panama	NY
Meriania_costata_AD106	MH781569	MH781618	MH819892	MH760326	MH781683	MH747607	Dellinger A. 106	Ecuador	QCNE, W
Meriania_crassiramis_T2944	no	MH781619	MH819893	MH760327	MH781684	MH747608	Radosavljevic A. 258	Guyana	NY, US

Meriania_cuzcoana_T2658	MH781570	MH781620	MH819894	MH760328	MH781685	MH747609	Michelangeli F. A. 1908	Peru	NY, USM
Meriania_denticulata_DF64	no	no	MH819895	no	no	no	Homeier H. 2202	Ecuador	NY
Meriania_drakei_aff3_AD153	MH781571	MH781621	MH819896	MH760329	MH781686	MH747610	Dellinger A. 153	Ecuador	QCNE, W
Meriania_drakei_drakei_AD142	MH781572	MH781622	MH819897	MH760330	MH781687	MH747611	Dellinger A. 142	Ecuador	QCNE, W
Meriania_ekmanii_T2664	KJ933884	KJ933925	KJ933972	MH760331	KJ934025	MH747612	Majure L.C. 4299	Haiti	FLAS
Meriania_fantastica_MA1851	no	no	KY991960	MF105460	MF104929	no	Alvear M. 1851	Colombia	COL, CAS
Meriania_franciscana_CU1795	KF820114	KF820878	KF821539	MH760332	KF822064	MH747613	Ulloa C.U. 1795	Ecuador	MO
Meriania_furvanthera_AD23	MH781573	MH781623	MH819898	MH760333	MH781688	no	Dellinger A. 23	Ecuador	QCNE, W
Meriania_grandiflora_DSP1746	MG198496	MF029525	KY991550	MF105649	no	MF069964	Penneys D. S. 1746	Panama	FLAS
Meriania_haemantha_FA10546	no	MF029216	KY991700	MF105360	MF104794	no	Almeda F. 10546	Colombia	COL, CAS
Meriania_haemantha_v_orientalis_FA10651	KF819889	KF820609	KF821418	KY991658	MF104808	no	Almeda F. 10651	Colombia	COL, CAS
Meriania_hernandoi_FA10300	MG198234	MF029174	KY991658	MF105337	MF104758	MF069669	Almeda F. 10300	Colombia	COL, CAS
Meriania_hexamera_AD139	no	MH781624	MH819899	MH760334	MH781689	MH747614	Dellinger A. 139	Ecuador	QCNE, W
Meriania_hexamera_T1680	KF820115	KF820879	KF821540	MH760335	KF822065	MH747615	Ulloa C.U. 1825	Ecuador	MO
Meriania_inflata_T2786	MH781574	MH781625	MH819900	MH760336	MH781690	MH747616	Goldenberg R. 2078	Brazil	UPCB
Meriania_involucrata_T270	KF820116	KF820880	EF418874	MF105734	KF822066	MF070034	Skean D. 4097	Dom. Rep.	FLAS
Meriania_kirkbridei_DF48	no	no	MH819901	no	no	no	Fernandez D. M. 1541	Ecuador	QCN
Axinaea_lawsonnonii_cf_AD116	MH781575	MH781626	MH819902	MH760337	MH781691	MH747617	Dellinger A. 116	Ecuador	QCNE, W
Meriania_leucantha_T1695	KF820117	KF820881	KF821541	MH760338	KF822067	MH747618	Judd W.S. 8303	Jamaica	FLAS
Meriania_longifolia_FA10169	MG198207	MF029145	KY991629	MF105298	MF104711	MF069630	Almeda F. 10169	Colombia	COL, CAS
Meriania_longifolia_FAM610	JQ730316	KF820882	AY460454	no	KF822068	no	Michelangeli F.A. 610	Venezuela	BH, VEN
Meriania_loxensis_AD115	MH781576	MH781627	MH819903	MH760339	MH781692	MH747619	Dellinger A. 115	Ecuador	QCNE, W
Meriania_macrophylla_costanensis_FAM829	KF820118	KF820883	AY460455	no	KF822069	no	Michelangeli F.A. 829	Venezuela	BH, VEN
Meriania_macrophylla_macrophylla_DSP1741	MG198495	MF029524	KY991549	MF105647	MF105164	MF069962	Penneys D. S. 1741	Panama	NY
Meriania_maguirei_AD110	no	MH781628	MH819904	MH760340	MH781693	no	Dellinger A. 110	Ecuador	QCNE, W
Meriania_maxima_DSP1618	MG198486	MF029505	KY991539	MF105617	MF105138	MF069946	Penneys D. S. 1618	Ecuador	FLAS
Meriania_mexiae_DSP1848	no	no	KY991554	no	no	MF069974	Penneys D. S. 1848	Ecuador	NY
Meriania_nobilis_MEM1781	MG198468	MF029474	KY991969	no	no	no	Morales M. E. 1781	Colombia	UPTC

Meriania_nobilis_T2767	MH781577	MH781629	MH819905	no	MH781694	no	Clark J. L. 13051	Colombia	UNA
Meriania_panamensis_DSP1734	MG198493	MF029521	KY991546	MF105644	no	MF069960	Penneys D. S. 1734	Panama	FLAS
Meriania_paniculata_T3022	MH781578	MH781630	MH819906	no	MH781695	no	Reginato M. 1477	Brazil	NY, UPCB
Meriania_parvifolia_T2300	KJ933885	KJ933926	KJ933973	MH760341	KJ934026	MH747620	Skean D. 5048	Haiti	FLAS
Meriania_pastazana_AD143	no	no	MH819907	MH760342	MH781696	MH747621	Dellinger A. 143	Ecuador	QCNE, W
Meriania_peltata_AD148	no	MH781631	MH819908	MH760343	MH781697	MH747622	Dellinger A. 148	Ecuador	QCNE, W
Meriania_phlomoides_FA10354	MG198254	MF029196	KY991680	MF105353	MF104777	MF069687	Almeda F. 10354	Colombia	COL, CAS
Meriania_pichichensis_DSP1905	MG198506	MF029556	KY991563	MF105685	MF105197	MF069982	Penneys D. S. 1905	Ecuador	NY
Meriania_purpurea_T1696	KF820119	KF820885	KF821542	MH760344	KF822071	MH747623	Judd W.S. 8306	Jamaica	FLAS
Meriania_quintuplinervis_FA10306	MG198237	MF029177	KY991661	MF105340	MF104761	MF069672	Almeda F. 10306	Colombia	COL, CAS
Meriania_radula_cf_AD126	no	MH781632	MH819909	MH760345	MH781698	MH747624	Dellinger A. 126	Ecuador	QCNE, W
Meriania_radula_T2008	MH781579	MH781633	MH819910	MH760346	MH781699	MH747625	Michelangeli F. A. 1732	Peru	NY, USM
Meriania_rigida_aff_T3056	MH781580	no	MH819911	no	MH781700	no	Michelangeli F.A. 2635	Peru	NY, USM
Meriania_rigida_DSP1617	MG198485	MF029504	KY991538	MF105616	MF105137	MF069945	Penneys D. S. 1617	Ecuador	FLAS
Meriania_robusta_T1717	KF820120	KF820886	no	no	KF822072	no	Michelangeli F.A. 1623	Brazil	NY, UPCB
Meriania_rugosa_T2006	MH781581	MH781634	MH819912	MH760347	MH781701	MH747626	Michelangeli F. A. 1704	Peru	NY, USM
Meriania_sanguinea_aff_T3087	MH781582	MH781635	MH819913	MH760348	MH781702	MH747627	Michelangeli F.A. 2743	Peru	NY, USM
Meriania_sanguinea_DSP1588	MG198481	no	KY991534	MF105611	no	no	Penneys D. S. 1588	Ecuador	NY
Meriania_sanguinea_T3338	MH781583	no	no	MH760349	MH781703	MH747628	Fernandez D. M. 2215	Ecuador	QCN
Meriania_sclerophylla_T706	KF820121	KF820887	KY991910	no	KF822073	MF069919	Redden K.M. 1219	Guyana	NY, US
Meriania_selvaflorensis_MA1465	MG198326	MF029324	KY991930	no	MF104901	MF069778	Alvear M. 1465	Colombia	COL, CAS
Meriania_silverstonei_FA10348	MG198252	MF029193	KY991677	MF105352	MF104774	MF069684	Almeda F. 10348	Colombia	COL, CAS
Meriania_sp_AD149	no	MH781636	MH819914	MH760350	MH781704	MH747629	Dellinger A. 149	Ecuador	QCNE, W
Meriania_sp_AD155	no	MH781637	MH819915	MH760351	MH781705	MH747630	Dellinger A. 155	Ecuador	QCNE, W
Meriania_sp_FA10147	MG198203	MF029140	KY991623	no	MF104705	MF069624	Almeda F. 10147	Colombia	COL, CAS
Meriania_sp_FA10184	MG198211	MF029148	KY991633	MF105301	MF104714	MF069634	Almeda F. 10184	Colombia	COL, CAS
Meriania_sp_faldas_AD146	no	MH781638	MH819916	MH760352	MH781706	MH747631	Dellinger A. 146	Ecuador	QCNE, W
Meriania_sp_MA1475	MG198327	MF029325	KY991931	MF105437	MF104902	no	Alvear M. 1475	Colombia	COL, CAS

Meriania_sp_T2915	MH781584	MH781639	MH819917	no	MH781707	no	Michelangeli F.A. 1991	Peru	NY, USM
Meriania_speciosa_FA10219	MG198216	MF029156	KY991640	MF105308	MF104722	MF069640	Almeda F. 10219	Colombia	COL, CAS
Meriania_splendens_MA1690	no	MH781640	MH819918	MH760353	MH781708	MH747632	Alvear M. 1690	Colombia	COL, CAS
Meriania_spnov_AD157	no	MH781641	MH819919	MH760354	MH781709	MH747633	Dellinger A. 157	Ecuador	QCNE, W
Meriania_squamulosa_T2665	KJ933886	KJ933927	KJ933974	MH760355	KJ934027	no?	Skean D. 5053	Haiti	FLAS
Meriania_steyermarkii_FAM1266	MG198457	MF029462	KY991809	MF105577	MF105104	MF069903	Michelangeli F. A. 1266	Venezuela	NY, VEN
Meriania_subumbellata_FAM819	KF820122	KF820889	AY460457	MH760356	KF822075	MH747634	Michelangeli F.A. 819	Venezuela	BH, VEN
Meriania_tetragona_AD107	MH781585	MH781642	MH819920	MH760357	MH781710	MH747635	Dellinger A. 107	Ecuador	QCNE, W
Meriania_tetragona_T2009	no	MH781643	MH819921	MH760358	MH781711	MH747636	Michelangeli F. A. 1739	Peru	NY, USM
Meriania_tetramera_T972	no	MH781644	MH819922	MH760359	MH781712	MH747637	Goldenberg R. 911	Brazil	UPCB
Meriania_tomentosa_aff_AD144	MH781586	MH781645	MH819923	MH760360	MH781713	MH747638	Dellinger A. 144	Ecuador	QCNE, W
Meriania_tomentosa_DSP1899	MG198505	MF029553	KY991562	MF105682	no	MF069981	Penneys D. S. 1899	Ecuador	FLAS
Meriania_tomentosa_T3051	no	MH781646	MH819924	no	MH781714	no	Michelangeli F.A. 2623	Peru	NY, USM
Meriania_tuberculata_T2274	MH781587	MH781647	MH819925	MH760361	MH781715	MH747639	Pedraza P.P. 2142	Colombia	NY
Meriania_urceolata_FAM539	KF820124	KF820891	AY460458	no	KF822077	MH747640	Michelangeli F.A. 539	Venezuela	BH, VEN
Meriania_weberbaueri_T3078	MH781588	MH781648	MH819926	MH760362	MH781716	MH747641	Michelangeli F.A. 2714	Peru	NY, USM
Miconia calycina	KF820179	KF820956	EU055737	EU056001	KF822139	JF832003	Judd, W., 8210 (FLAS)	Puerto Rico	FLAS
Physeterostemon_fiaschii_T319	KF820526	KF821337	KF821756	EU711379	KF822520	EU711397	Amorim A.M. 4515	Brazil	CEPEC
Physeterostemon_jardimii_T742	KF820527	KF821338	KF821757	EU711382	KF822521	EU711399	Amorim A.M. 7064	Brazil	CEPEC
Physeterostemon_thomasii_T355	JQ730332	KF821339	KF821758	EU711383	JQ730542	EU711401	Amorim A.M. 5054	Brazil	CEPEC
Salpinga_glandulosa_T2938	MH781589	MH781649	MH819927	no?	MH781717	no	Goldenberg R. 1941	Brazil	UPCB
Salpinga_maranoensis_JLC6979	MG198373	MF029372	KY991873	JF831982	MF104983	JF832008	Clark J. L. 6979	Ecuador	NY
Salpinga_peruviana_T_3331_T3336	MH781590	MH781650	MH819928	MH760363	MH781718	MH747642	Clark J. L. 15100	Ecuador	UNA
Salpinga_secunda_FAM487	MG198459	no	KY991815	EU711384	MF105092	EU711402	Michelangeli F. A. 487	Peru	NY, USM
Tococa guianensis	KF820567	KF821385	AY460554	EU056136	KF822559	AM235650	Michelangeli, F. A., 703 (BH)	Venezuela	BH

Table S6. Predictive value of floral characters used in traditional pollination syndromes (e.g. Ollerton et al. 2009, Lagomarsino et al. 2016) in Merianieae (measured by reduction in Gini index), the floral traits belonging to the 20 most important floral characters identified are marked in bold.

Traditional pollination syndrome characters in Merianieae	Reduction in Gini index	Relative ranking
**Reward type**	0.802	**2**
Positioning of inflorescence	0.060	38
**Flower orientation**	0.624	**3**
Maximal corolla opening	0.141	23
**Corolla height**	0.490	**9**
**Corolla shape**	0.492	**7**
Corolla colour	0.122	25
**Petal gloss**	0.600	**5**
Scent	0.109	26
Arrangement of androecium relative to corolla	0.098	29
**Level of anther pore relative to style**	0.356	**15**
**Adaxial thecal wall**	0.368	**14**
Colour contrast appendage/thecae	0.059	44
**Relation between stigma and corolla**	0.622	**4**
Timing of anthesis	not included	not included

Table S7. Estimated average number of pollination syndrome shifts across 1000 stochastic character mappings, the total average number of pollination syndrome transitions is 10.675.

ancestral syndrome	shifted syndrome	average number of shifts
buzz-bee	mixed-vertebrate	3.402
buzz-bee	passerine	5.839
mixed-vertebrate	buzz-bee	0.468
mixed-vertebrate	passerine	0.277
passerine	buzz-bee	0.501
passerine	mixed-vertebrate	0.188

Table S8. Results from post-hoc test on morphological differences between pollination syndromes (Bonferroni corrected, PERMANOVA). F value is given in the upper part of each classification method, * indicates significant p-value 0.01667.

	buzz-**bee**	**MV**	**pass**
buzz-**bee**		34.389	25.717
**MV**	*		49.674
**pass**	*	*	

Table S9. Results from post-hoc test on significant differences in disparity (mean pairwise differences) between pollination syndromes. * indicates p-value < 0.001.

	buzz-**bee**	**MV**	**pass**
buzz-**bee**		2.985	7.862
**MV**	0.0085		3.971
**pass**	*	*	

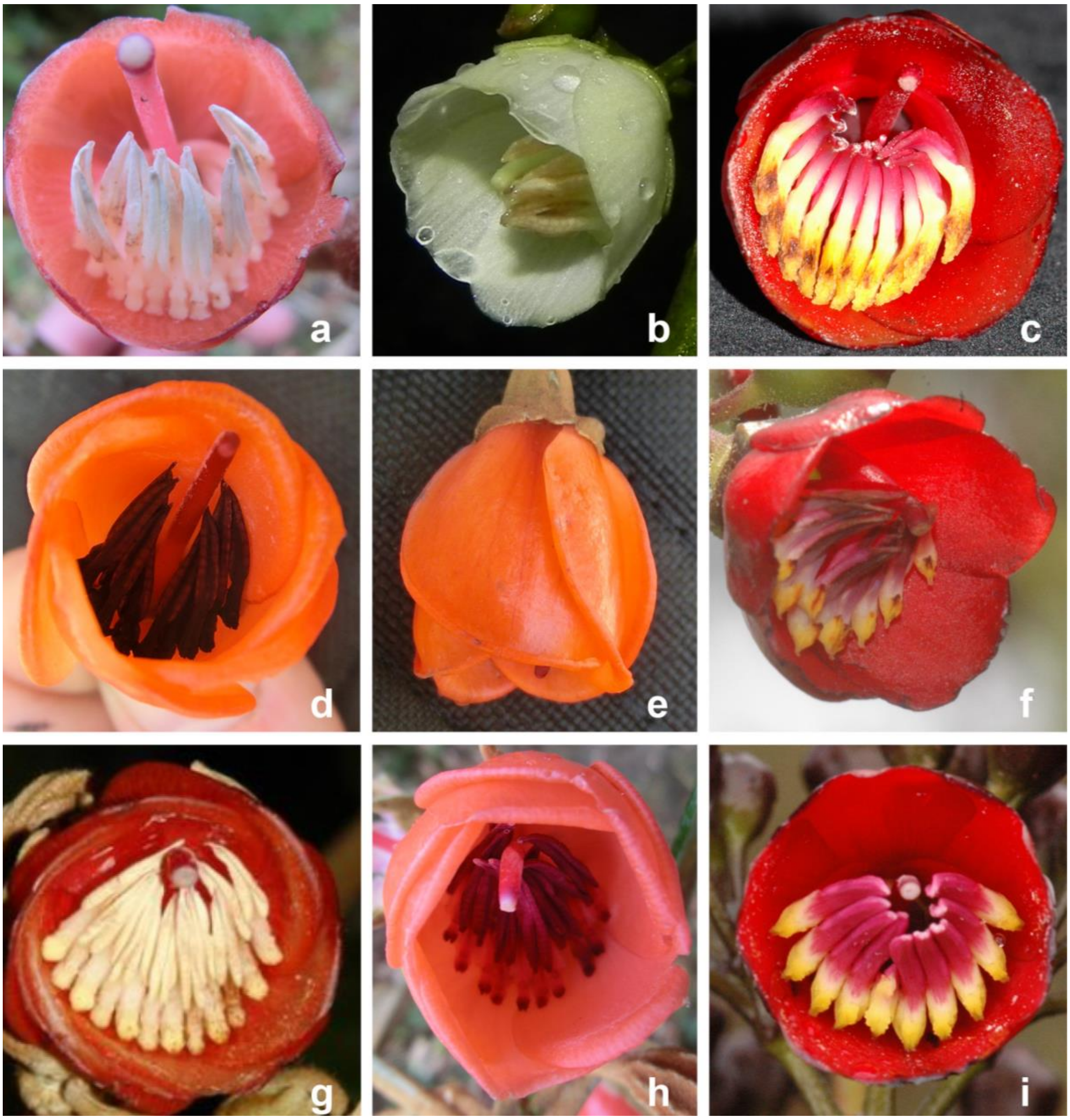

Figure S1. Nectar producing *Meriania* species with known pollinators grouped into the 'mixed vertebrate' pollination syndrome. a-c: hummingbird/bat pollinated, (a) *M. tomentosa*, (b) *M. phlomoides*, (c) *M.* aff. *sanguinea*. d, e: flowerpiercer/rodent pollinated *M. furvanthera*. f: hummingbird/rodent pollinated *M. sanguinea*. g: hummingbird pollinated *M. quintuplinervis*, night observations have never been done. h: hummingbird pollinated *M. costata*, night observations have never been done. i: *M. tetragona*, hummingbirds observed close to flowers, night observations have never been done. Given the large similarity of g, h, i, to species where both day and night monitoring was conducted and both diurnal (hummingbirds, flowerpiercers) and nocturnal (bats, rodents) pollinators were observed, nocturnal pollinator visits in g, h, i are highly probable.

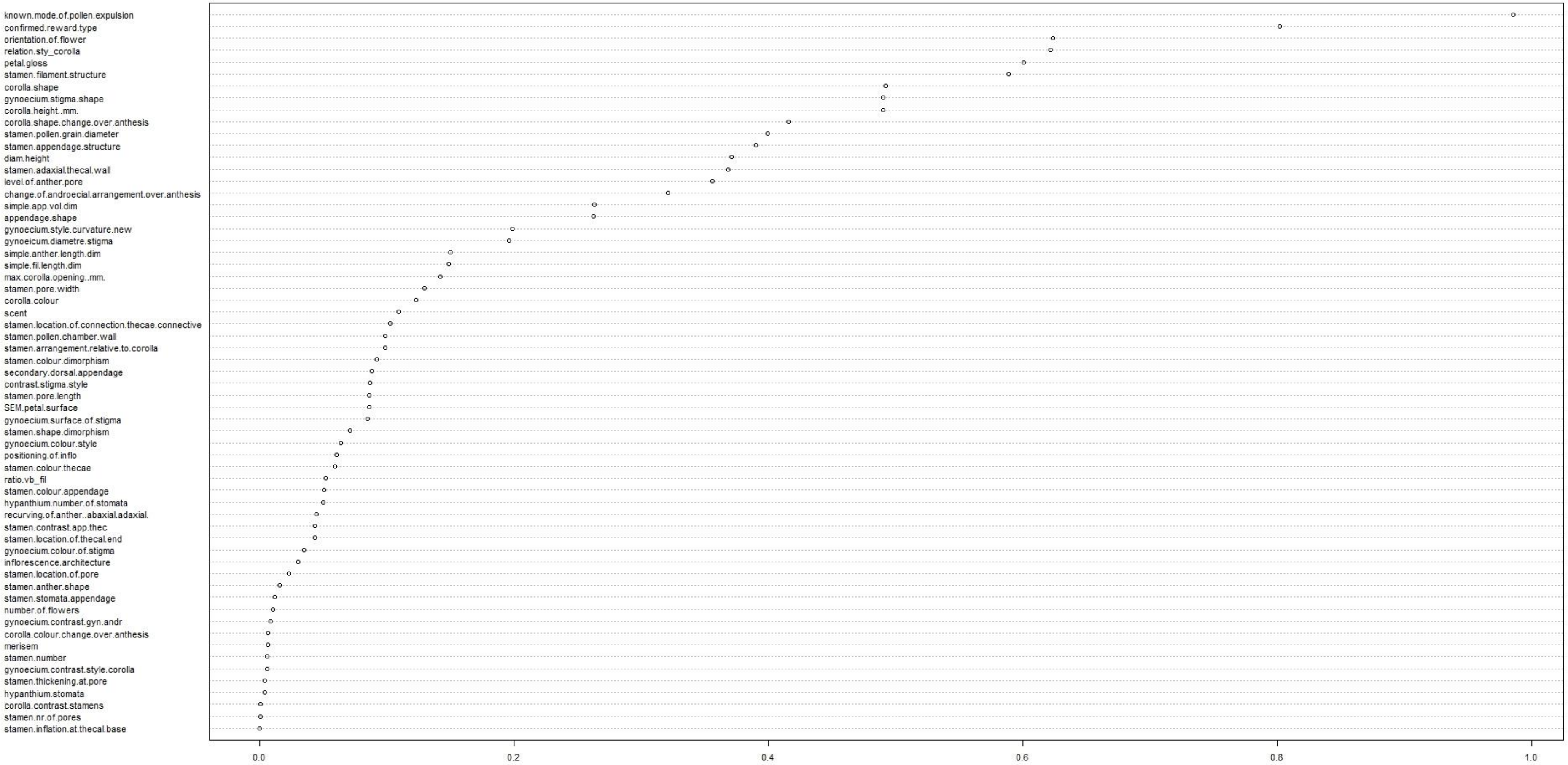

Figure S2. Ranking of all 61 assessed floral characters based on their importance in predicting pollination syndromes in Merianieae (based on Gini Index).

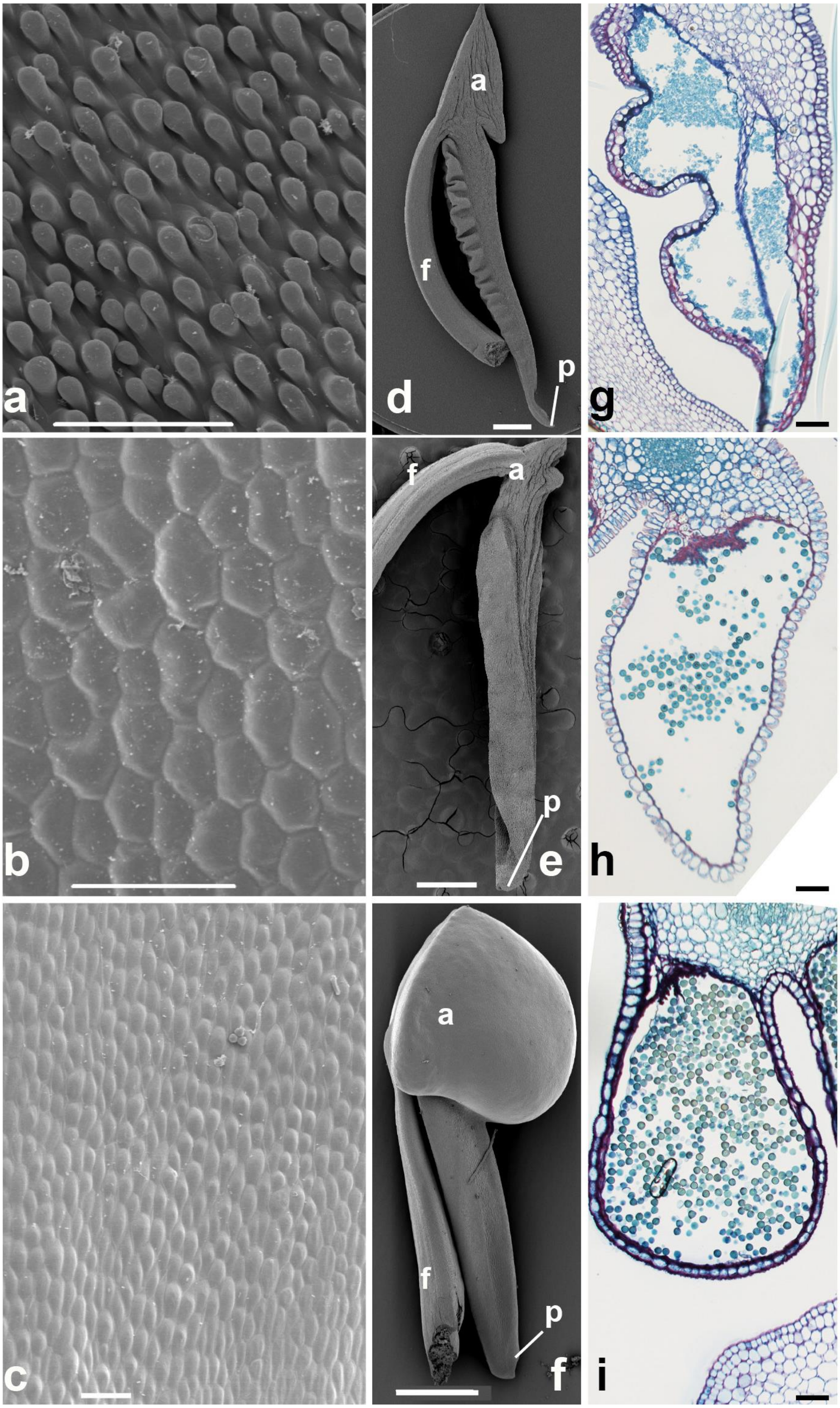
a
d
a
f
p
g
b
f
a
p
e
h
c
a
f
p
f
i

Figure S3. Structural properties of petals and stamens in Merianieae. (a) 'buzz-bee' syndrome petal surface with papillate epidermis of *Meriania brachycera*. (b) 'Mixed vertebrate' syndrome petal surface with almost smooth epidermis of *M. tomentosa*s (c) 'Passerine' syndrome petal surface with smooth epidermis of *Axinaea costaricensis*. (d) 'buzz-bee' syndrome stamen of *M. haemantha* ssp. *haemantha,* note ventral attachment of corrugated thecae to connective and sculptured appendage (e) 'Mixed vertebrate" syndrome stamen of *M. furvanthera*, note lateral attachment of pollen chambers to connective and small appendage. (f) 'Passerine' syndrome stamen of *Axinaea costaricensis* with bulbous appendage and ventral attachment of pollen chambers to connective. (g) Cross-section of theca of 'buzz-bee' syndrome *M. haemantha* ssp. *haemantha*, note epidermis and endothecium with thickened cell walls as well as corrugated structure of thecal wall and presence of septum separating the two pollen sacs of the theca. (h) Cross-section of theca of 'mixed vertebrate' syndrome *M. pichichensis* with flexible pollen chamber wall and collapsed septum (remnants indicated with arrowhead). (i) Cross-section of theca of 'passerine' syndrome *A. costaricensis* with smooth thecae with thickened cell walls in epidermis and collapsed septum (arrowhead). a – appendage, f – filament, p – pore, scale bars: (a), (b), (g), (h), (i), (j) 100 µm; (c), (l) 200 µm; (k) 500 µm; (d), (e), (f) 1 mm.

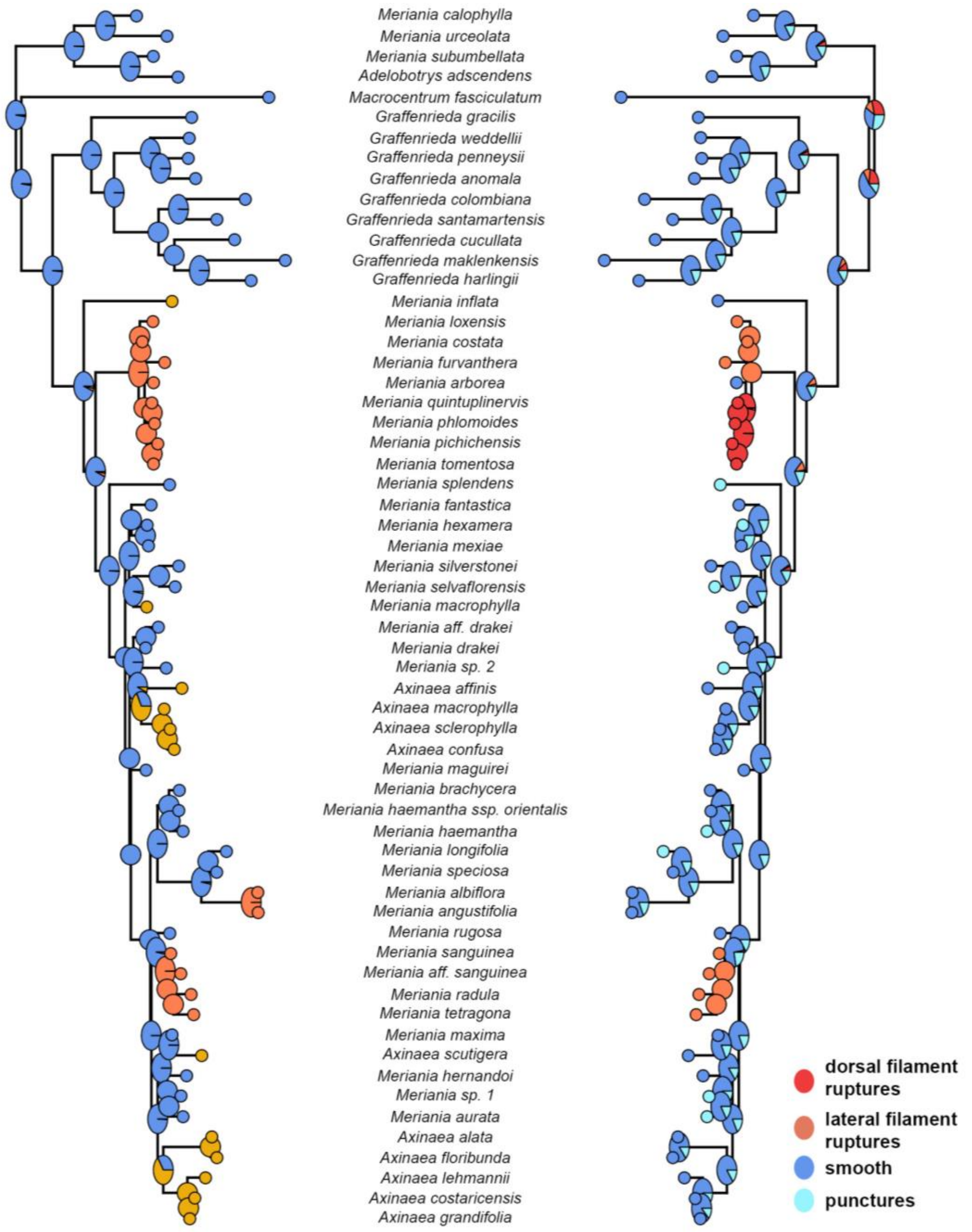

Figure S4. Stochastic character mapping of pollination syndromes (left) and the 'filament structure' (right). Note that filament ruptures are only found within the 'mixed-vertebrate' syndrome (in salmon on the left) while the ancestral 'buzz-bee' syndrome (blue on the left) and the 'passerine' syndrome (yellow on the left) do not show filament ruptures. The 'all rates different' model was chosen to estimate filament structure evolution as it performed significantly better than the 'equal rates' model (ER: log-likelihood: -53,5, AIC 109, ARD: log-likelihood: -36.6, AIC 97, ANOVA: $p < 0.001$).

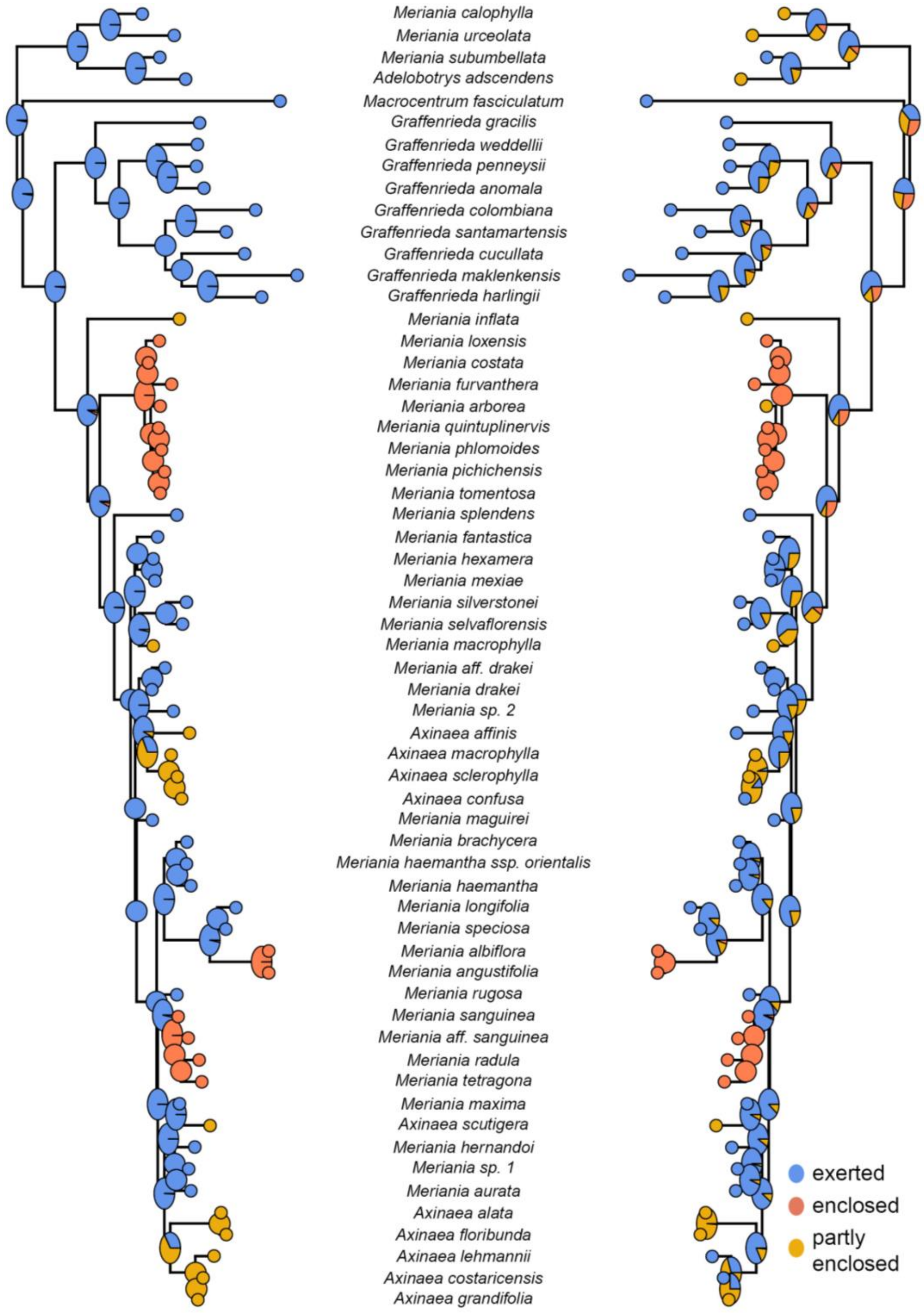

Figure S5. Stochastic character mapping of pollination syndromes (left) and the character 'relation style to corolla' (right). Note that in all 'mixed-vertebrate' species (in salmon on the left), styles are enclosed by the pseudo-campanulate corolla, while 'passerine' syndrome species (in yellow on the left) have more open corollas with only partly enclosed or exerted styles and most 'buzz-bee' syndrome flowers (in blue on the left) have fully exerted styles ('ER' model: log-likelihood -47.2, AIC96.4, 'ARD' model. Log-likelihood -40.1, AIC 92.3, ANOVA p 0.014).

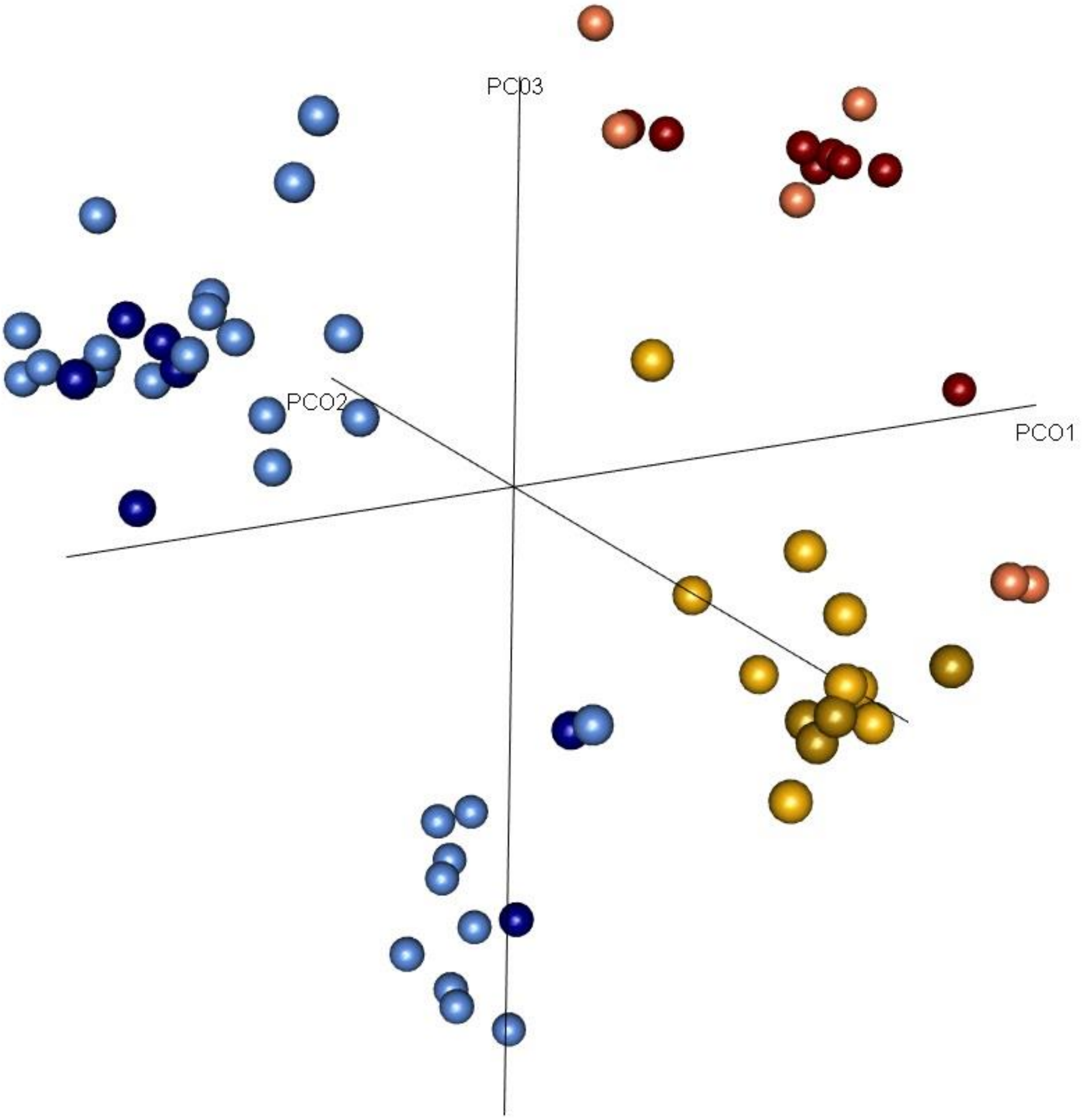

Figure S6. Merianieae morphospace PC1-3. The three pollination syndromes (‘buzz-bee’ – blue, ‘mixed-vertebrate’ – red, ‘passerine’ – yellow) are clearly differentiated; species with known pollinators are represented in darker colours while lighter colours represent species estimated into syndromes by RF analyses. Note the large disparity of buzz-bee pollinated species and the three distinct clusters found within the ‘buzz-bee’ syndrome.

Notes S1. 61 floral characters coded for Merianieae and used to evaluate pollination syndromes in the tribe

Descriptions of characters and decision criteria for character states are given. Characters relevant for understanding flower functioning and pollination biology in Merianieae were targeted while not focusing on characters only relevant for taxonomic treatments (justification of character choices are given in brackets). These floral characters could be used for the inclusion of further taxa within the tribe, but should mostly also be applicable to other Melastomataceae tribes.

1. **Reward type** (traditional pollination syndrome character)
   0) Pollen
   1) Nectar
   2) Food body
2. **Inflorescence architecture** – evaluated on photos, herbarium specimens and in the field, following description of inflorescences by Cotton et al. 2014 (possibly relevant for how pollinators can approach flowers; Harder & Prusinkiewicz, 2013)
   0) Compound or simple dichasium, subtended by a pair of leaf-like bracts, p. 14, Cotton et al. 2014, p.14, Figure 3C and D
   1) Elongate thyrse, elongated inflorescence with bracts absent or caduceus or occasional small leaf-like bracts, Cotton et al. 2014, p.14, Figure 3B
   2) Elongate whorls (whorls along an extended inflorescence stalk like e.g. *M. sanguinea*)
   3) Leafy snyflorescence, subtended by successively smaller pairs of leaf-like bracts, Cotton et al. 2014, p.14, Figure 3A
3. **Number of flowers** – evaluated on photos, herbarium specimens and in the field, following Cotton et al. 2014 (possibly relevant for floral display)
   0) Few (1-10 flowers per inflorescence)
   1) Moderate (11-25 flowers per inflorescence)
   2) Rich (>26 flowers per inflorescence)
4. **Position of inflorescence in relation to foliage** – evaluated on photos, herbarium specimens and in the field (possibly relevant for how pollinators can approach flowers)
   0) Not projected
   1) Projected (flowers clearly extended from foliage e.g. by an elongated inflorescence stalk or terminal positioning in vine (*Adelobotrys*), easily visible)
5. **Orientation of flowers in inflorescence** - evaluated on photos and herbarium specimens and considering the majority of flowers (traditional pollination syndrome character)
   0) Multiple
   1) Upright-horzontal
   2) Nodding
6. **Merisem** – evaluated on photos, herbarium specimens and in the field; if individuals with variable merosity were present, the most common condition was coded unless different types of merosity were equally abundant (an increase in merisem was mostly observed in bee pollinated species)

0) 4
1) 5
2) 6
3) 5 - 7

7. **Hypanthial stomata** – assessed on hypanthia prepared for SEM (the hypanthium has been proposed as site of nectar secretion (Varassin et al. 2008))
   0) Yes
   1) No
8. **Number of stomata** in 1/10th of the hypanthium counted on samples prepared for SEM (numeric, 0-349); (the hypanthium has been proposed as site of nectar secretion (Varassin et al. 2008))
9. **Maximal corolla opening** – maximal opening of petal tips, measured on 3D-models of flowers in AMIRA (numeric (mm)); (traditional pollination syndrome character, flower size)
10. **Corolla height** – measured on longitudinal sections of 3D-models of flowers in AMIRA from the hypanthium rim to the highest point of the corolla (numeric (mm)); (traditional pollination syndrome character)
11. **Ratio between corolla diameter (9) and corolla height (10)** – numeric (traditional pollination syndrome character, indicative of flower shape or tube width)
12. **Corolla shape** - assessed at mid-anthesis (thus excluding opening buds (which at first will all resemble cupule/funnel shapes) and senescent flowers (which will have opened more in certain species)), evaluated on photos and pickled material (traditional pollination syndrome character, important for fit with pollinator and physical restriction of flower access in many other plant lineages)
   0) Bowl-shaped without overlapping margins (*Axinaea*s with corolla more widely open)
   1) Bowl shaped to flat (Meriania species)
   2) Campanulate (bell-shaped, pendant corollas)
   3) Campanulate-salverform (slightly campanulate with reflexed petal tips)
   4) Solanum type (*Graffenrieda*; similar to *Solanum*-type flower with central circle of stamens and reflexed petals)
   5) Urceolate *(Axinaea*s, bell-shaped flowers with an opening narrower than the maximum corolla diameter)
13. **Corolla shape change** over anthesis - estimated on photos, in the field and on pickled material (this could potentially change the accessibility to rewards (e.g. in a pseudo-campanulate flower, large bees could be limited in finding optima buzzing positions)
   0) Weak (hardly any change/some spreading of the corolla but only within a shape category)
   1) Strong (i.e. change from one shape category to another (e.g. from cupule to basin))
14. **Corolla colour change over anthesis** - evaluated on photos and in the field (could influence pollinator attraction, compare Brito et al. 2015)
   0) No
   1) Yes
15. **Corolla colour** - evaluated on photos and in the field, using X-rite Colour Checker as a reference (traditional pollination syndrome character)

0) White
1) cream pink
2) Red
3) Salmon
4) Fuchsia
5) Orange
6) Lilac

16. **Colour contrast between corolla and stamens** – based on photos (traditional pollination syndrome character, important for pollinator attraction)
    0) Yes
    1) No
17. **Petal gloss** - evaluated on flowers in the field and if high quality photos were available (traditional pollination syndrome character, pollinator attraction)
    0) Matt
    1) Gloss
18. **Petal surface** - SEM was used to assess the shape of epidermis cells on the ventral petal surface (with bee pollinated flowers usually having conical cells (mostly long papillate, enhancing grip and visibility), and bird pollinated flowers usually having flat surface cells (see Papiorek et al. 2014 for more details))
    0) Smooth
    1) Short papillate
    2) Long papillate
19. **Scent** – evaluated in the field (smelling with the human nose; traditional pollination syndrome character, pollinator attraction)
    0) Flowery
    1) Heavy-sweet
    2) No
    3) Weak (if not all test persons could perceive a smell, but 50% claimed to smell something)
20. **Number of stamens** – evaluated on photos and observations of pickled material (an increase in stamen number was mostly observed in bee pollinated species)
    0) 8
    1) 10
    2) 12
    3) 10-14
21. **Stamen shape dimorphism** – evaluated on photos and observations of pickled material (heteranthery is known to be an important trait in buzz-pollination (Vallejo-Marín et al., 2010)
    0) Isomorphic
    1) slightly dimorphic (small differences in shape or size, but no heteranthery)
    2) strongly dimorphic (heteranthery)
22. **Dimorphism in filament length** – evaluated on pickled material (heteranthery is known to be an important trait in buzz-pollination (Vallejo-Marín et al., 2010)
    0) Yes (if filaments bring the two stamen whorls to different heights)
    1) No

23. **Dimorphism in appendage volume** – evaluated on pickled material (heteranthery is known to be an important trait in buzz-pollination (Vallejo-Marín et al., 2010)
    0) Yes
    1) No
24. **Dimorphism in anther length** – evaluated on pickled material (heteranthery is known to be an important trait in buzz-pollination (Vallejo-Marín et al., 2010)
    0) Yes
    1) No
25. **Stamen colour dimorphism** – evaluated on photos and in field (heteranthery is known to be an important trait in buzz-pollination (Vallejo-Marín et al., 2010)
    0) Yes
    1) No
26. **Stamen arrangement relative to corolla -** the corolla is divided into 5 sections (following the petals in pentamerous species, extrapolating this pattern in hexa- and heptamerous species) and stamen arrangement is classed into these 5 sections by evaluating how many fifth are covered by the appendage tips, evaluated on pickled material and photos (possibly relevant for where the pollinator positions itself on the flower)
    0) 2/5
    1) 3/5
    2) 4/5
    3) 5/5
    4) 3/4
27. **Level of anther pore -** height of the anther pores relative to the style length (measured from style base), evaluated on pickled material (determines site of pollen release in relation to other floral organs)
    0) Top (anther pores close to stigma)
    1) Middle (anther pores located higher than 1/3 of style length but lower than 90% of style length)
    2) Bottom (anther pores located close to style base)
    3) Top/middle (in strongly dimorphic species)
28. **Change of androecial arrangement over anthesis** – evaluated on pickled material, photos and in field (possible change of site of pollen release)
    0) No – androecium remains more or less constant in position during anthesis
    1) Weak – irregular spreading during anthesis
    2) Strong – strong reflexive movement of stamens and migration of pores towards stigma during anthesis
29. **Secondary dorsal stamen appendage shape** – evaluated on pickled material (stamen appendages are sites of interaction with the pollinator (to obtain the reward) at least in bee and passerine pollinated species (Renner 1989, Dellinger et al. 2014))
    0) Bifurcate (bifurcated, often elongated)
    1) Knob (protrusion bending upwards (away from connective strand, not towards pore (compare “nose”)), sitting on connective strand; found in *M. tomentosa* group)

2) Nose (rounded structure bending towards pore, sitting on connective strand; found e.g. in *M. haemantha*)
3) Absent (no secondary appendage present)

30. Shape of primary stamen appendage – evaluated on pickled material (stamen appendages are sites of interaction with the pollinator (to obtain the reward) at least in bee and passerine pollinated species (Renner 1989, Dellinger et al. 2014))
0) Acuminate (*Graffenrieda*; small spine, separate from thecae)
1) Bulbous-acuminate (*M. macrophylla*)
2) Bulbous (in *Axinaea*, similar width:length, ratio 0.5 to > 1)
3) Crown (severals *Merianias*, similar to pyramidal but ending in a rugged tip (instead of an acuminate one))
4) Fusiform (elongated, width:length < 0.25; more direct transition into thecae)
5) Pyramidal (triangular acuminate pyramid, width:length > 0.33, including species with more distant thecae (e.g. *M. sanguinea* but also *M. haemantha ssp haemantha*)

31. Known mode of pollen expulsion – evaluated in the field by pollinator observations and experimental manipulation using tweezers (to mimick birds' bills, compare Dellinger et al. 2014) and tuning forks (to mimick buzzing bees)
0) Buzzing
1) Bellows-mechanism
2) Salt-shaker like pollen release

32. Location of thecae on connective – evaluated on pickled material (location is related to the mechanism of pollen release, pollen is released more easily on laterally attached thecae)
0) Ventral (thecae restricted to dorsal side of connective strand)
1) Lateral (thecae attached at sides of connective strand, pollen chambers supinated)

33. Location of thecal end (end of pollen chambers) in relation to appendage – evaluated on pickled material (possibly related to pollen release)
0) Base (thecae end at appendage base, actual end of pollen chamber often only visible in cross-sections)
1) Offset (thecae end a few mm/cm away from appendage base, only connective strand reaches appendage base)

34. Anther shape – evaluated on pickled material (possibly related to pollen release/pollen dosing)
0) Acuminate (continuous narrowing towards the pore, width at pore considerably less than on top)
1) Oblong (oblong anther which only narrows just before the pore but remains more or less the same thickness)
2) Acuminate/oblong (dimorphic stamens)

35. Recurving of anther - curvature from adaxial to abaxial side (to differentiate more or less straight, cannon-like anthers from curved anthers (mostly at the apex); careful, this should not be confused with anthers elevated due to reflexion of the filament), evaluated on pickled material (possibly related to pollen release/pollen dosing)
0) Yes

1) No

36. Spatulate broadening of thecae around anther pore – evaluated using SEM (possibly related to pollen release/pollen dosing)

0) Yes
1) No

37. Structure of adaxial thecal wall – evaluated on pickled material and SEM (possibly related to pollen release/pollen dosing)

0) Ruminate (sturdy and strongly folded, made up by more than one tightly arranged cell layer (possibly a remaining)
1) Smooth (sturdy but NOT folded, made up by one tightly arranged cell layer and strong cuticle and remnants of tapetum)
2) Crumpled (soft and flexible, made up by one more loosely arranged cell layer)

38. Thecae separated into two pollen sacs by septum– evaluated on cross sections of stamens using microtome sectioning/light microscopy and cross-sections of stamens of HRXCT-scans of flowers in AMIRA (possibly related to pollen release/pollen dosing)

0) Yes
1) No
2) Reduced wall between pollen sacs (in some *Graffenrieda* species)

39. Number of stamen pores– evaluated on SEM (possibly related to pollen release/pollen dosing)

0) 1
1) 2
2) 1 or 2 (rare, found in some strongly heterantherous species)

40. Location of pore on anther– evaluated on SEM (possibly related to pollen release/pollen dosing)

0) Apical (the pore is strictly apical with no inclination)
1) Dorsal (the pore is on the dorsal side with a lip hindering pollen from flying into the apical direction)
2) Dorsal/Apical (in some strongly heterantherous species, stamen whorls differ in the inclination of the pore)
3) Dorsal tip (the pore is dorsally inclined but mostly opens to the front, the lip (compare with dorsal) is lacking)
4) Ventral (the pore is ventrally inclined)

41. Pore width – 10 stamens/species measured on 3D models of flowers in AMIRA, mean taken (numeric (mm)); (possibly related to pollen release/pollen dosing)

42. Pore height – 10 stamens/species measured on 3D models of flowers in AMIRA, mean taken (numeric (mm)); (possibly related to pollen release/pollen dosing)

43. Pollen grain diameter – 10 pollen grains/species measured in 70% ethanol using a fluorescence microscope, mean taken (numeric (mm)); (possibly related to pollen release/pollen dosing)

44. Structure of stamen filaments – filaments have been found to constitute the location of nectar secretion, evaluated using light microscopy and SEM; (filament ruptures have been detected as sites of nectar secretion (Dellinger et al., unpublished data))

0) Dorsal ruptures (necrotic horizontal slits on the dorsal side)

1) Small intercellular holes on proximal lateral side of filament and/or rupture on filament/connective joint
2) Smooth
3) Punctures (rounded necrotic surface damages; down to vascular bundle in some species)

45. **Structure of stamen appendage surfaces**– evaluated on SEM (appendage surface structures may influence the grip for pollinators applying vibrations)
    0) Smooth (no protrusions or groves)
    1) Smooth-pitted (generally smooth, but some depressions)
    2) Cauliflower (both horizontal and vertical grooves, like cauliflower)
    3) Mixed-bumpy (in M. *tomentosa*-group, appendages that have features of suclate/cauliflower but also smooth parts and a generally bumpy surface)
    4) Sulcate (mainly vertical grooves but overall even surface (without cauliflower protrusions)
    5) Papillate (papillae on appendage)
46. **Inflation at thecal base** – evaluated on SEM (possibly related to pollen release/pollen dosing)
    0) Yes
    1) No
47. **Stomata on stamen appendage**– evaluated on SEM (these could potentially be related to nectar or scent emission, Varassin et al., 2008, Dellinger et al., unpublished data)
    0) No
    1) Occasional (sometimes up to five)
    2) Regular (more than five in all stamens)
48. **Ratio vascular bundle:filament width** – numeric (measured on sections of CT-scans, 5 stamens per specimen, at the base of the filament; coronal plane); (thick vascular bundles have been detected in nectar releasing Melastomataceae by Varassin et al., 2008)
49. **Colour stamen appendage** (traditional pollination syndrome character, visual attraction)
    0) Colour appendage
    1) Cream
    2) Yellow
    3) Blue
    4) Fuchsia
    5) Dark violet
50. **Colour thecae** (traditional pollination syndrome character, visual attraction)
    0) Cream
    1) Yellow
    2) White
    3) Red
    4) fuchsia
    5) Dark violet

51. **Colour contrast thecae and stamen appendage** – evaluated on photos and in field (traditional pollination syndrome character, visual attraction)
    0) Yes
    1) No
52. **Relative position of style and corolla** – evaluated on pickled material, viewed from the front/side (traditional pollination syndrome character, related to fit between flower and pollinator)
    0) Free (style usually visible in its full length)
    1) Partly enclosed (upper quarter of the style usually visible)
    2) Enclosed (style mostly enclosed by petals, not (or only tip of stigma) visible)
53. **Style curvature** – evaluated on pickled material (possibly governs pollen pick-up from pollinator; e.g. a hooked style would only pick up pollen if the pollinator positioned itself directly underneath)
    0) Curved (variable curvature, slightly curved to almost straight in 90% of flowers)
    1) Hooked (strong hook at tip in > 90% of flowers)
54. **Stigma diameter** – measured on 3D scans of flowers, mean taken (numeric (mm)); (possibly related to pollen pick-up, Cruden 2000)
55. **Stigma shape** - interpreted when placing the style upright and looking at the stigma from the side in SEM (possibly related to pollen pick-up)
    0) Corymbose (umbrella-shape, overarching the width of the style but usually shorter than wide, sometimes almost rounded like a ball)
    1) Convex (bump, shorter than wide, but not overarching style width)
    2) Conical (elongated, as long or longer than wide, not overarching style width)
    3) Stamp (almost flat, about as wide as the style, neither narrowing nor widening)
56. **Stigma surface** - evaluated on SEM (possibly related to pollen pick-up)
    0) Densely papillate (papillae heads attach closely to each other)
    1) Scarcely papillate (space between papillae)
57. **Colour of style** – evaluated on photos and in the field (visual attraction)
    0) White
    1) Light pink
    2) Fuchsia
    3) Red
    4) Lilac
    5) Salmon
58. **Colour of stigma** – evaluated on photos and in the field (visual attraction)
    0) White
    1) Light pink
    2) Fuchsia
    3) Red
    4) Lilac
    5) Dark purple
59. **Colour contrast style – corolla** – evaluated on photos and in the field (visual attraction)
    0) No

1) Yes
2) Weak

60. Colour contrast androecium – gynoecium – evaluated on photos and in the field (visual attraction)

0) No
1) Yes
2) Weak

61. Colour contrast between stigma and style – evaluated on photos and in the field (visual attraction)

0) No
1) Yes

Notes S2. Detailed description of Merianieae pollination syndromes

Bee syndrome flowers in Merianieae are characterized by a pollen reward, which is released by high-frequency buzzes applied by bees to the stamens. Flowers are often upright or horizontally oriented with wide bowl-shaped to deflexed corollas, with a mean diameter:height ratio of 8.7. Corolla shape changes markedly in the first hours/day of anthesis when corollas gradually reflex. Petal epidermis cells were found to be conical in shape. Flower colours range widely from white to different shades of pink and lilac, with stamens usually forming a strong colour contrast. Stamens may be arranged either on one side of the flower, giving the flowers a distinct monosymmetric architecture (*Meriania, Adelobotrys, Macrocentrum*), or the stamens are distributed more or less regularly in the flower, leading to almost polysymmetric flowers (*Graffenrieda*). Anthers can be erect (*Graffenrieda*), bringing pores close to the stigma, or remain geniculate (the condition found in bud-stage in all species) with pores remaining close to the base of the style in the floral centre. Stamen appendages are usually very conspicuous and variable in shape, pyramidal to weakly acuminate, sometimes bearing secondary appendages, and often have strongly ornamented surfaces. Weak to strong heteranthery is found in all *Adelobotrys* and some *Meriania* species. Thecae are located on the ventral side of the connective and usually have strongly corrugated and rigid walls consisting of two cell layers and an endothecium. A septum separating the thecae into two pollen sacs is present. Pores may be located on the dorsal (*Meriania*, partly *Adelobotrys*) or ventral (*Graffenrieda*, *Macrocentrum*) side of the anther. Styles are usually exserted from the rest of the flower and often strongly curved right beneath the stigma. In many species, stigmas are small and punctiform. Flowery, pleasant scents have been noticed in some species in *Meriania* and *Adelobotrys* (ASD pers. obs.). Anthesis usually starts in the early morning and may last from a single to multiple days (ASD pers. obs.). Bees have been observed in four large flowered *Meriania* species orientating their bodies in parallel to individual stamens, with their head at the appendage and their abdomen pointing towards the pores. They bite into the appendage and vibrate individual stamens at a time. In smaller flowered *A. adscendens*, bees were seen to crouch above the entire androecium (instead of single stamens), head pointing towards the flower centre, and applying vibrations to the entire androecium. Thus, the bee-syndrome encompasses various types of interactions between flowers and buzzing bees.

Flowers belonging to the ‘MV’ syndrome provide nectar rewards secreted from the stamens and aggregating on the petals (Dellinger et al., unpublished). Flowers are usually pendant and

pseudo-campanulate, with a diameter:height ratio of 1.0. Petal epidermis cells are usually flat, petals glossy and colours range from white, pinkish, salmon to scarlet red. All species have androecia arranged on one side of the flower and stamens undergoing a strong deflexion movement in the early phase of anthesis, bringing pores close to stigmas (anthers erect). Stamen appendages are smaller than in bee-pollinated *Meriania* species, crown shaped and relatively inconspicuous in colouration in some species (e.g., hummingbird/bat pollinated *M. tomentosa*), but larger and more vividly coloured in others (e.g., hummingbird/rodent pollinated *M. sanguinea*). Heteranthery is absent in most of these species, it is present, however, in the Antillean *M. angustifolia* and *M. albiflora*, both of which showed considerable inconsistency in pollination syndrome assignment (alternative: bee; see below). In many species, thecae are attached laterally to the connective. They have a soft, easily deformable (e.g. by a hummingbird's bill) wall made up of the epidermis only. The septum separating the thecae has collapsed. Apical anther pores are usually directed towards the stigma. Styles are often straight, not exceeding the corolla length, and often bear enlarged, slightly flattened stigmas. Floral scent can range from scentless (for the human nose, e.g. *M. furvanthera*) to emitting a flowery perfume-like scent (e.g. *M. tomentosa*) or strong, glue/plastic-like scents in *M. sanguinea* (for details see Dellinger et al., unpublished). Flowers become anthetic in mornings and/or evenings and usually remain open for approximately three days. Mixed diurnal and nocturnal pollinator assemblages have been observed drinking nectar in five species. When the animals insert their bills or tongues/heads into the pseudo-campanulate corollas, they push through the densely arranged anthers to lick nectar aggregated beneath the stamens. They thereby touch the soft, laterally attached thecae and cause pollen release. As all stamens are arranged with the pores pointing downwards, out of the pendant flower, this mechanism is termed 'salt-shaker' like pollen release.

The passerine pollination syndrome is characterized by staminal food body rewards, which at the same time function as pollen expulsion mechanism ('bellows'-mechanism). Passerine syndrome flowers are usually oriented in various directions (upright, horizontal, pendant) with mostly urceolate corollas with a diameter:height ratio of 1.5, which does not change much during anthesis in most species (compare with 'bee' syndrome). Petal epidermis cells were flat to slightly conical and petals were matte matt, colours range from light pink to red, and yellow corollas are also known. In all species with passerine pollination, the brightly coloured stamen appendages form a strong colour contrast with the corolla. Stamens are arranged on one side of the flower (monosymmetric) and in contrast to the 'MV' syndrome, they do not

deflex during anthesis so that the pores remain more or less around the mid length of the style. All species are united by characteristic bulbous stamen appendages with smooth surfaces. Most species show moderate heteranthery mostly in appendage volume and colour. Only *Meriania macrophylla* has strongly dimorphic stamens, a trait otherwise only found in the 'bee' syndrome (see estimation results below). Thecae are located on the ventral side of the connective and have a smooth, sturdy wall, composed of the epidermal cell layer and an endothecium. As in the 'MV'-syndrome, the septum has collapsed. Pores are located on the dorsal side of the anther. Styles are usually partially exserted from the urceolate corollas, with relatively small, conical stigmas. No scents have been noticed with the human nose (ASD, pers. obs.). Anthesis starts in the early morning and lasts for several days up to a week (ASD, pers. obs.). Passerines (tanagers, flowerpiercers) have been observed feeding on the bulbous stamen appendages in three species. The appendages contain high amounts of sugars (food body reward) and also function as a pollen expulsion mechanism: when passerines bite the appendages for consumption, the compression forces contained air into and through the thecae, dusting the birds with pollen grains that are ejected out of the apical pores.

References

Brito VLG, Weynans K, Sazima M, Lunau K. 2015. Trees as huge flowers and flowers as oversized floral guides: the role of floral color change and retention of old flowers in *Tibouchina pulchra.* Frontiers in Plant Sciences **6**: 362.

Caleron-Saenz E. 2012. Cultivo de melastomatáceas con potencial ornamental en reservas naturales de la sociedad civil. Reserva Natural “El Refugio”.

Cotton E, Borchsenius F, Balslev H. 2014. *A revision of Axinaea (Melastomataceae). Sci Dan B Biol* **Vol 4**. Det Kongelige Danske Videnskabernes Selskab, 120 pp.

Cruden RW. 2000. Pollen grains: why so many? Plant Systematics and Evolution **222**: 143-165.

Dellinger AS, Penneys DS, Staedler YM, Fragner L, Weckwerth W, Schönenberger J. 2014. A Specialized Bird Pollination System with a Bellows Mechanism for Pollen Transfer and Staminal Food Body Rewards. *Current Biology* **24**: 1615–1619.

Harder LD, Prusinkiewicz P. 2013. The interplay between inflorescence development and function as the crucible of architectural diversity. Annals of Botany **112**: 1477-1493.

Lagomarsino LP, Forrestel EJ, Muchhala ND, Charles C. 2017. Repeated evolution of vertebrate pollination syndromes in a recently diverged Andean plant clade. *Evolution* **71**(8): 1970–1985. doi: 10.1111/evo.13297.

Muchhala N, Jarrin-V P. 2002. Flower Visitation by Bats in Cloud Forests of Western Ecuador. *Biotropica* **34**: 387–395.

Ollerton J, Alarcón R, Waser NM, Price MV, Watts S, Cranmer L, Hingston A, Peter CI, Rotenberry J. 2009. A global test of the pollination syndrome hypothesis. *Annals of Botany* **103**(9): 1471–1480.

Papiorek S, Junker RR, Lunau K. 2014. Gloss, Colour and Grip: Multifunctional Epidermal Cell Shapes in Bee- and Bird-Pollinated Flowers. *PLOS ONE* **9**(11): e112013.

Renner SS. 1989. A survey of reproductive biology in Neotropical Melastomataceae and Memecylaceae. *Annals of the Missouri Botanical Garden* **50**: 496–518.

Rojas-Nossa SV. 2007. Estrategias de extracción de néctar por pinchaflores (aves: *Diglossa* y *Diglossopis*) y sus efectos sobre la polinización de plantas de los altos andes. *Ornitología Colombiana* **5**: 21-39.

Vallejo-Marín M, Da Silva EM, Sargent RD, Barrett SC. 2010. Trait correltes and functional significance of heteranthery in flowering plants. New Phytologist **188**: 418-425.

Varassin IG, Penneys DS, Michelangeli FA. 2008. Comparative Anatomy and Morphology of Nectar-producing Melastomataceae. *Annals of Botany* **102**: 899–909.
